# Clinical Characterization and Outcomes of Human Clade IIb Mpox Virus Disease: A European Multicenter Mpox Observational Cohort Study (MOSAIC)

**DOI:** 10.1093/cid/ciae657

**Published:** 2025-01-03

**Authors:** Elise Pesonel, Cédric Laouénan, Laetitia Guiraud, Josephine Bourner, Isabelle Hoffmann, Diana Molino, Coralie Tardivon, Delphine Bachelet, France Mentré, Alain Amstutz, Laura Merson, Amanda Rojek, Minerva Cervantes Gonzalez, Andrea Antinori, Antonella Castagna, Silvia Nozza, Valérie Pourcher, Agnès Libois, Jake Dunning, Evelina Tacconelli, Maya Hites, Fernando De La Calle Prieto, Peter Horby, Yazdan Yazdanpanah, Alexandra Calmy, François-Xavier Lescure, Piero Olliaro, for the Mpox ObServAtIonal Cohort (MOSAIC) Study Group, Maya Hites, Leïla Belkhir, Marie-Angélique De Scheerder, Jean-Christophe Goffard, Agnès Libois, Zineb Khalil, Catherine Orban, Lucie Seyler, Clotilde Visée, Florence Ader, Antoine Bachelard, Yasmine Bouaraba, Jean-Marc Chapplain, Hugues Cordel, François Danion, Aurélien Dinh, Nikita Dobremel, Manuel Etienne, Pierre Frange, Karine Faure, François Goehringer, Karine Lacombe, Xavier Lescure, Rodolphe Manaquin, Guillaume Martin-Blondel, Jean-Michel Mansuy, Giovanny Gombert, Duc Nguyen, Romain Palich, Gilles Pialoux, Aoife Cotter, Virginie Gautier, Evelina Tacconelli, Oriana Awwad, Andrea Antinori, Antonella Castagna, Silvia Nozza, Angelo Roberto Raccagni, Elena Bruzzesi, Antonio Cascio, Annamaria Cattelan, Maddalena Cordioli, Giulia De Luca, Lorenza Lambertenghi, Giulia Marchetti, Valentina Mazzotta, Emanuele Nicastri, Vincenzo Scaglione, Carlo Tascini, Jorge Machado, Lurdes Santos, Wong Chen Seong, Travis Ren Teen Chia, Wilnard Yeong Tze Tan, Fernando De La Calle Prieto, Vicente Estrada, Miguel Nicolás Navarrete Lorite, Alfredo Soler Carracedo, Maria Velasco Arribas, Dominique Braun, Alexandra Calmy, Matthias Cavassini, Lorenzo Ciullini, Stéphane Emonet, Chiara Fedeli, Yvan Gosmain, Laetitia Guiraud, Benjamin Hampel, Olivier Segeral, Cornelia Staehelin, Marcel Stöckle, Alejandro Arenas-Pinto, Mike Beadsworth, Margherita Bracchi, Joby Cole, Jake Dunning, Michael Marks, Brendan Payne, Malcolm G Semple, Claire Waddington, Chris Ward, Alain Amstutz, Dominique Costagliola, Jérémie Guedj, Inge Christoffer Olsen, Diane Descamps, Christophe Batejat, Jessica Vanhomwegen, Maude Bouscambert-Duchamp, Julio Garcia Rodriguez, Alexandre Gaymard, Andreas Lind, Fabrizio Maggi, Hervé Raoul, Jeroen Van Kampen, Sabine Yerly, Nicolas Yin, Léo Chenard, Ismaila Deme, Alpha Diallo, Séverine Gibowski, Sabrina Kali, Guillaume Le Meut, Sophie Letrou, Claire Madelaine, Hervé Raoul, Ventzislava Petrov Sanchez

**Affiliations:** International Severe Acute Respiratory and Emerging Infection Consortium–Pandemic Sciences Institute, University of Oxford, Oxford, United Kingdom; Department of Epidemiology Biostatistics and Clinical Research, Assistance Publique–Hôpitaux de Paris (AP-HP) Nord, Hôpital Bichat, Paris, France; Infections, Antimicrobials, Modelling, Evolution (IAME) Research Centre, Unité mixte de recherche(UMR) 1137,Université Paris Cité, Institut national de la santé et de la recherche médicale (INSERM), Paris, France; HIV/AIDS Unit, Division of Infectious Diseases, Geneva University Hospitals, Geneva, Switzerland; International Severe Acute Respiratory and Emerging Infection Consortium–Pandemic Sciences Institute, University of Oxford, Oxford, United Kingdom; Department of Epidemiology Biostatistics and Clinical Research, Assistance Publique–Hôpitaux de Paris (AP-HP) Nord, Hôpital Bichat, Paris, France; INSERM, National Agency for Research on AIDS and Viral Hepatitis–Emerging Infectious Diseases, Paris, France; Department of Epidemiology Biostatistics and Clinical Research, Assistance Publique–Hôpitaux de Paris (AP-HP) Nord, Hôpital Bichat, Paris, France; Department of Epidemiology Biostatistics and Clinical Research, Assistance Publique–Hôpitaux de Paris (AP-HP) Nord, Hôpital Bichat, Paris, France; Department of Epidemiology Biostatistics and Clinical Research, Assistance Publique–Hôpitaux de Paris (AP-HP) Nord, Hôpital Bichat, Paris, France; Infections, Antimicrobials, Modelling, Evolution (IAME) Research Centre, Unité mixte de recherche(UMR) 1137,Université Paris Cité, Institut national de la santé et de la recherche médicale (INSERM), Paris, France; Division of Clinical Epidemiology, Department of Clinical Research, University Hospital Basel and University of Basel, Basel, Switzerland; Oslo Center for Biostatistics and Epidemiology, Oslo University Hospital, Oslo, Norway; Population Health Sciences, Bristol Medical School, University of Bristol, Bristol, United Kingdom; International Severe Acute Respiratory and Emerging Infection Consortium–Pandemic Sciences Institute, University of Oxford, Oxford, United Kingdom; International Severe Acute Respiratory and Emerging Infection Consortium–Pandemic Sciences Institute, University of Oxford, Oxford, United Kingdom; Department of Epidemiology Biostatistics and Clinical Research, Assistance Publique–Hôpitaux de Paris (AP-HP) Nord, Hôpital Bichat, Paris, France; Infections, Antimicrobials, Modelling, Evolution (IAME) Research Centre, Unité mixte de recherche(UMR) 1137,Université Paris Cité, Institut national de la santé et de la recherche médicale (INSERM), Paris, France; National Institute for Infectious Diseases Lazzaro Spallanzani, Scientific Institute for Research, Hospitalization and Healthcare (IRCCS), Rome, Italy; Infectious Diseases Unit, Vita-Salute University, Milan, Italy; Infectious Diseases Unit, IRCCS, San Raffaele Scientific Institute, Milan, Italy; Infectious Diseases Unit, Vita-Salute University, Milan, Italy; Infectious Diseases Unit, IRCCS, San Raffaele Scientific Institute, Milan, Italy; Infectious Diseases Department, Sorbonne University, Pitié-Salpêtrière Hospital, AP-HP, Pierre-Louis Epidemiology and Public Health Institute, INSERM U1136, Paris, France; Department of Infectious Diseases, Centre hospitalier universitaire Saint-Pierre, Université Libre de Bruxelles, Brussels, Belgium; International Severe Acute Respiratory and Emerging Infection Consortium–Pandemic Sciences Institute, University of Oxford, Oxford, United Kingdom; Infectious Diseases, Department of Diagnostic and Public Health, University of Verona, Italy; Hôpital Universitaire de Bruxelles-Érasme, Université Libre de Bruxelles, Brussels, Belgium; National Reference Centre for Imported Diseases and International Health, High-Level Isolation Unit, La Paz–Carlos III University Hospital, CIBERINFEC, Madrid, Spain; International Severe Acute Respiratory and Emerging Infection Consortium–Pandemic Sciences Institute, University of Oxford, Oxford, United Kingdom; INSERM, National Agency for Research on AIDS and Viral Hepatitis–Emerging Infectious Diseases, Paris, France; HIV/AIDS Unit, Division of Infectious Diseases, Geneva University Hospitals, Geneva, Switzerland; Infections, Antimicrobials, Modelling, Evolution (IAME) Research Centre, Unité mixte de recherche(UMR) 1137,Université Paris Cité, Institut national de la santé et de la recherche médicale (INSERM), Paris, France; Infectious and Tropical Diseases Department, AP-HP, Bichat Hospital, Paris, France; International Severe Acute Respiratory and Emerging Infection Consortium–Pandemic Sciences Institute, University of Oxford, Oxford, United Kingdom

**Keywords:** mpox, clade IIb, viral load, lesion resolution, observational cohort

## Abstract

**Background:**

The global mpox outbreak that started in May 2022 was caused by a novel clade IIb variant of the mpox virus (*Orthopoxvirus monkeypox*, MPXV). It differed from the traditional Western and Central Africa disease in transmission patterns and clinical presentation.

**Methods:**

To address the need for detailed clinical and virologic data, we conducted an observational cohort study (MOSAIC) during May 2022–July 2023 in individuals with confirmed MPXV infection enrolled in 6 European countries. Case management decisions were left to the attending physician. Participants were monitored for up to 6 months for clinical signs/symptoms and clinical and virologic outcomes through hospital visits, phone interviews, and self-administered questionnaires. Outcomes included time to lesion resolution, clinical status, and virus clearance.

**Results:**

The 518 participants not receiving any specific treatment (“untreated”) were diagnosed a median 5 days from symptom onset; 90% were managed as outpatients. Lesions were mostly cutaneous (88%) and perigenital (74%). By day 14 from the first polymerase chain reaction (PCR)–positive sample, 39% had resolved lesions. Time to 95% unculturable virus was longest in cutaneous lesions (52 days). A putative systemic antiviral was available for 57 participants, 44% as inpatients; 34% and 58% had resolved lesions by day 14 from the first PCR-positive sample and from treatment start, respectively. Time to 95% unculturable virus was 60 days in skin and oropharynx. No death or recrudescence occurred by day 180.

**Conclusions:**

MOSAIC provides comprehensive insights into the clinical and virologic characteristics of mpox caused by the clade IIb variant. The study forms the basis of clinical characterization for ongoing mpox outbreaks.

## Teaser

In an observational study of 575 mpox cases from 6 European countries, 39% of untreated participants (90% managed as outpatients) had lesions resolved 14 days from laboratory-confirmed infection; skin lesions still had culturable virus in 5% of participants by day 52.

## Graphical Abstract

**Graphical Abstract:**
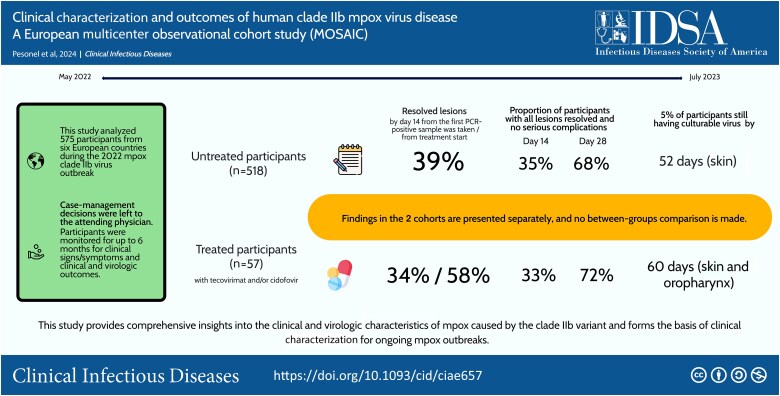
This graphical abstract is also available at Tidbit: https://tidbitapp.io/institutional-portal/clinical-infectious-diseases/tidbits/clinical-characterisation-and-outcomes-of-human-clade-iib-mpox-virus-disease-a-european-multicentre-observational-cohort-study-mosaic/update

Between July 2022 and May 2023, the World Health Organization (WHO) declared a Public Health Emergency of International Concern (PHEIC) for a multicountry outbreak of mpox caused by a novel clade IIb variant of the mpox virus (*Orthopoxvirus monkeypox*, MPXV). This outbreak was substantially different from the typical Central and Western Africa outbreaks in terms of transmission patterns. It marked the first large-scale global mpox event and the first time with extensive, sustained human-to-human transmission. The clinical presentations were also different: Rather than a rash covering most of the body, often with nonspecific symptoms including fever, fatigue, and lymphadenopathy [1], clade IIb MPXV appeared milder, patients typically presented with localized rashes [2, 3], and intimate contact was the primary route of transmission, in adults usually associated with sexual activity [3].

While knowledge was rapidly generated on clinical characterization and outcomes from retrospective case series [4–8], a need was recognized to establish a protocolized, multicountry evaluation of the clinical characteristics and outcomes of clade IIb mpox infection. To respond to this need, following a request from the European Medicines Agency (EMA) Emergency Task Force, we initiated a multicountry observational cohort study in May 2022 [9, 10], with the primary objective of describing the clinical and virologic characteristics and outcomes of participants with laboratory-confirmed MPXV primarily in the European region.

## METHODS

### Study Design

The Mpox ObServAtIonal Cohort (MOSAIC) was designed as a multicountry observational cohort study to describe natural history, clinical presentation, and clinical and virologic outcomes of clade IIb MPXV disease—including both participants who were administered a systemic putative antiviral product and those who were not. MOSAIC was approved as an observational cohort study in the United Kingdom (UK), Switzerland, and Singapore, but was classified as a low-intervention clinical trial in participating European Union (EU) countries, according to EU Clinical Trial Regulation 536/2014.

MOSAIC enrolled inpatients and outpatients with either laboratory-confirmed MPXV infection or with clinically suspected mpox pending laboratory confirmation; those testing negative were subsequently withdrawn. Participants were enrolled at any time following diagnosis or suspicion of mpox and followed for up to 6 months. Treatment decisions were left to treating physicians according to national guidelines and drug availability.

Data on clinical signs and symptoms were collected on day (D) 1, D14, D28, D60, and D180 from the date the first sample was taken that returned a positive PCR result for MPXV. Data were also collected for all days of hospitalization for inpatients up to D180. Data could be collected retrospectively or prospectively, depending on the time elapsed between the collection of the diagnostic sample and the participant's consent. Data were collected via participant interview during hospital visits or by phone, as well as from the participant's hospital record or from participants’ responses to self-administered questionnaires.

Two lesion swab samples (skin or mucosal), 1 oropharyngeal swab sample, and 1 blood sample were to be collected on D1, D4, D8, D14 and D28, as permitted by local and national infection prevention and control measures.

The data dictionary is available in Supplementary Appendix 1. All data were recorded on REDCap (version 13.1.30) [11, 12].

### Setting

MOSAIC was approved in 51 hospitals in 7 countries: Belgium, France, Italy, Singapore, Spain, Switzerland, and the UK. Singapore did not enroll any participants.

### Outcomes

The primary outcome measure was time to lesion resolution (defined as the first day on which on which any of the following criteria were met: all lesions are resorbed, scabbed, or desquamated, and mucosal ulcers healed) without any serious complications by D14 (defined as a serious adverse event [SAE] that is life-threatening, results in hospitalization, prolongation of existing hospitalization, disability, incapacity, or a congenital anomaly; or any other medically significant complication).

Secondary outcome measures evaluated clinical and virologic outcomes. Clinical status was assessed on D14 and D28 according to a 4-point ordinal scale: (1) all lesions are resolved and no serious complications, (2) active lesions and no serious complications, (3) serious complication and/or hospitalization due to mpox, or (4) death.

Evidence of recrudescence or relapse was also assessed at D60 and D180 either in-person or remotely.

The number and type of SAEs, suspected adverse reactions, and suspected unexpected serious adverse reactions were assessed up to D28 and assessed by the study medical monitor.

Virologic outcomes were assessed on D4, D8, D14, and D28 and included changes in cycle threshold (Ct) value from baseline in blood, lesion, throat, and anal samples.

### Statistical Methods

Due to the descriptive nature of this study and the challenges in predicting the trajectory of case numbers during an outbreak, no formal sample size calculation was conducted.

The overall analysis population includes all participants who met the eligibility criteria and had at least 1 lesion at baseline.

Data and analyses are presented for the overall population and by treatment status. Participants who received an mpox-specific systemic antiviral product (defined as administration of systemic active antiviral treatment: tecovirimat, cidofovir, or tecovirimat plus cidofovir) within 14 days of their first mpox PCR-positive sample are classified as “treated participants.” Any other participant is classified as “untreated participants.”

Time to event analyses were done for all participants considering as baseline the date of the first MPXV PCR-positive sample. The same analyses were repeated for treated participants counting from the first day of antiviral treatment.

Time to lesion resolution is presented by treatment status using Kaplan-Meier (KM) curves until D28. KM estimates were used to compute the percentage and 95% confidence interval (CI) of participants with lesions resolved without complications at D14 and D28. Clinical status at D14 and D28 is presented as number and percentage of participants according to the above-mentioned 4-point ordinal scale for all eligible participants and according to treatment status.

Sankey diagrams summarizing change in clinical status from D14 to D28 are presented for treated and untreated participants who attended both follow-up visits. For treated participants, 2 Sankey diagrams are presented showing D14 and D28 from (1) treatment initiation and (2) the date the first sample was taken that returned a positive PCR result for mpox.

A linear mixed-effects model was used to describe the kinetics of the Ct values in mpox-infected individuals for each treatment group and to estimate the time to Ct ≥40 (indicative of virus undetectability) as well as the time to Ct ≥30 (used as a proxy for shedding of culturable virus) in plasma and skin, oropharyngeal, and rectal/perianal swabs (see Supplementary Methods for detailed statistical methods).

The probabilities of detectable and culturable virus were calculated by simulations (see Supplementary Methods for detailed statistical methods).

The Medical Dictionary for Regulatory Activities (MedDRA) System Organ Class and Preferred Terms are presented for each adverse event (AE) and SAE for the treated and untreated cohorts using the MedDRA Web-based browser version 27.0 [13]. Analyses were performed using R Statistical Software (version 4.1.2; R Core Team 2021).

### Virology Laboratory Methods

All procedures on MPXV infectious samples were conducted under strict biosafety conditions, according to national regulations and WHO recommendations. MPXV-specific real-time PCR assay was performed on samples using the local procedures available in each country. A PCR signal with Ct value ≥40 was considered negative.

### Ethics and Regulatory Approval

The study was registered on the EU Clinical Trials Register (2022-501132-42-00) and approved by the local ethics committees in each participating country (Supplementary Appendix 2).

## RESULTS

During June 2022–July 2023, 602 participants were enrolled in Europe across 37 hospitals out of the 51 outpatient and inpatient facilities that had been activated. Details of enrollment by country are reported in Supplementary Table 1.

**Figure 1.**
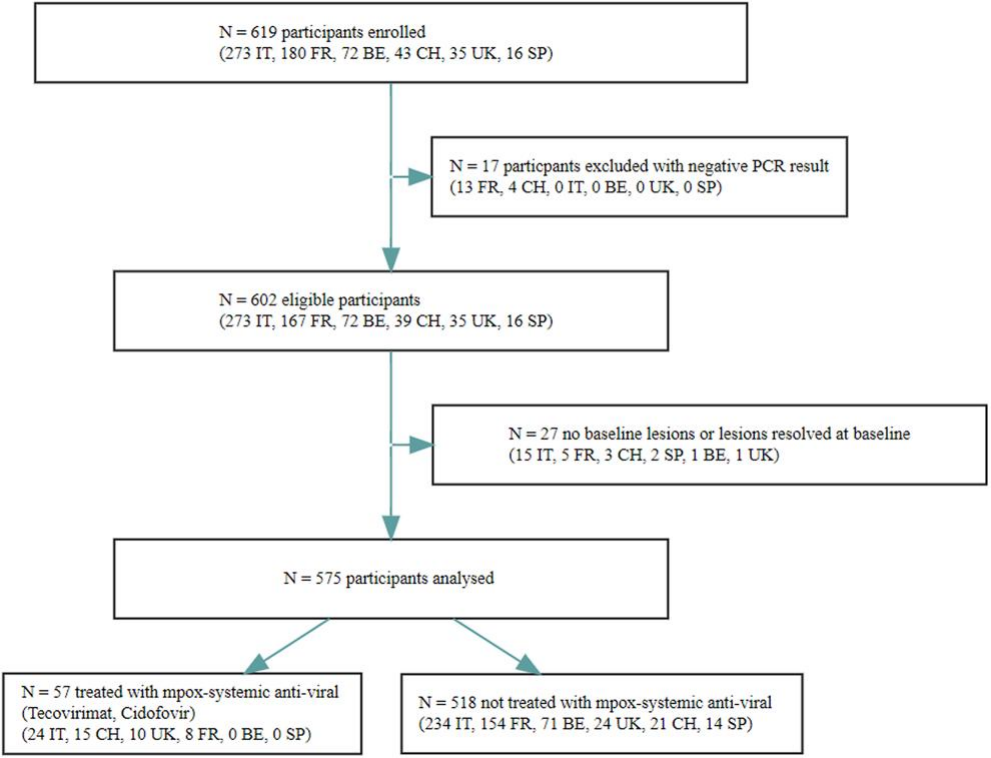
Flowchart summarizing study enrollment, eligibility, and inclusion in the analysis population. Abbreviations: BE, Belgium; CH, Switzerland; FR, France; IT, Italy; PCR, polymerase chain reaction; SP, Spain; UK, United Kingdom.

Of the participants enrolled, 27 were subsequently excluded from the analyses either because they had no lesions at baseline or because their lesions had already resolved (Figure 1). Of the 575 participants with unresolved lesions included in the final analysis, 57 (10%) were treated with a putative mpox-specific systemic antiviral (classified as “treated”) within 14 days of their initial presentation. As case management decisions were left to the attending physician and depended on drug availability, findings in the 2 cohorts are presented separately, and no between-groups comparison is made.

### Untreated Participants

Participants’ median age was 37 years (interquartile range [IQR], 32–44 years), and 99% were male (Table 1). One hundred six (22%) participants had a concomitant active sexually transmitted infection (STI), and 220 (43%) had a history of human immunodeficiency virus (HIV)/AIDS diagnosis, of whom 210 (95%) were on antiretroviral treatment; their median CD4 T-cell count at last follow-up was 743 cells/µL with 19 of 188 (10%) having CD4 <350 cells/μL. One hundred fifty three (51%) participants who did not have an HIV diagnosis were taking HIV pre- or postexposure prophylaxis.

**Table 1.** Baseline Demographic and Comorbidity Characteristics of Untreated and Treated Participants

Characteristic	Untreated (n = 518)	Treated (n = 57)
Demographics		
Age, y, median (IQR)	37 (32–44)	36 (32–43)
Female sex	5/515 (1)	1/57 (2)
Concomitant active STI	106/514 (22)	7/56 (12)
Syphilis	51/106 (48)	2/7 (29)
*Mycoplasma genitalium*	6/106 (6)	3/7 (43)
Chlamydia	19/106 (18)	0/7 (0)
Hepatitis B	3/106 (3)	0/7 (0)
HSV	7/106 (6)	0/7 (0)
Gonorrhea	33/106 (31)	0/7 (0)
HPV	2/106 (2)	0/7 (0)
*Ureaplasma*	3/106 (3)	3/7 (43)
Hepatitis C	5/514 (1)	0/56 (0)
HIV/AIDS	220/517 (43)	19/57 (33)
Receiving ARV	210/220 (95)	18/19 (95)
Viral load detectable	27/199 (14)	3/17 (18)
Viral load, copies/mL, median (IQR)	1480 (56–97 662)	84 (71–98)
CD4 T-cell count at last follow-up, cells/μL, median (IQR)	743 (530–1036)	652 (342–803)
Receiving HIV PrEP/PEP	153/297 (51)	12/38 (32)

Data are presented as No. (%) unless otherwise indicated.

Abbreviations: ARV, antiretroviral; HIV, human immunodeficiency virus; HPV, human papillomavirus; HSV, herpes simplex virus; IQR, interquartile range; PEP, postexposure prophylaxis; PrEP, preexposure prophylaxis; STI, sexually transmitted infection.

A median of 5 days (IQR, 3–8 days) had elapsed from symptom onset to the date when the PCR sample was taken for diagnosis; 90% were managed as outpatients (Table 2).

**Table 2.** Baseline Disease Characteristics of Untreated and Treated Participants

Characteristic	Untreated (n = 518)	Treated (n = 57)
Management after clinical presentation		
Outpatient	465/514 (90)	32/57 (56)
Admitted to hospital ward for isolation only	12/514 (2)	3/57 (5)
Admitted to hospital ward for clinical need	36/514 (7)	20/57 (35)
Intensive care unit admission	1/514 (0)	2/57 (4)
Median (IQR) days from:		
Symptom onset to PCR^+^ diagnosis	5 (3–8)	4 (2–8)
PCR^+^ diagnosis to enrollment	0 (0–0)	0 (0–3)
PCR^+^ diagnosis to treatment start	…	4 (3–5)
Symptoms		
Lymphadenopathy	243/506 (48)	40/55 (73)
Fever	239/508 (47)	30/54 (56)
Headache	103/511 (20)	18/54 (33)
Pharyngitis/tonsillitis	77/513 (15)	15/54 (28)
Lower respiratory symptoms	7/513 (1)	4/54 (7)
Ocular complications	4/513 (1)	6/54 (11)
Diarrhea/gastroenteritis	37/512 (7)	4/55 (7)
Nausea/vomiting	12/512 (2)	2/54 (4)
Encephalitis	1/512 (0.2)	1/54 (2)
Other complications		
Proctitis	131/511 (26)	11/55 (20)
Bacterial superinfection	21/316 (7)	8/29 (28)
Folliculitis/cellulitis	11/21 (52)	3/8 (38)
Bacteremia	0/21 (0)	1/8 (12)
Urinary tract	1/21 (5)	0/8 (0)
Other	9/21 (43)	4/8 (50)
Mpox lesions		
Skin/mucosal lesions		
Skin lesions only	255/511 (50)	25/56 (45)
Mucosal lesions only	61/511 (12)	5/56 (9)
Skin and mucosal lesions	195/511 (38)	26/56 (46)
Active skin lesion types		
Vesicle	226/293 (77)	31/37 (84)
Pustule	186/294 (63)	30/37 (81)
Ulcerated lesion	74/294 (25)	17/37 (46)
Hemorrhagic/bleeding lesions	4/232 (2)	1/25 (4)
Other	22/304 (7)	3/37 (8)
Lesion sites		
Head skin lesions	168/511 (33)	36/56 (64)
Ocular lesions within the orbit	1/511 (0)	1/56 (2)
Other skin lesions	266/512 (52)	37/56 (66)
Mucosal lesions	256/511 (50)	31/56 (55)
Perigenital or perianal lesions	380/511 (74)	42/56 (75)
Estimated total no. of active lesions		
1–5	237/450 (53)	10/51 (20)
6–25	175/450 (39)	24/51 (47)
26–100	26/450 (6)	13/51 (25)
>100	3/450 (1)	4/51 (8)
Pain score (out of 10), median (IQR)	6 (3–7)	6 (4–8)
Dichotomized pain score		
<6	27/66 (41)	8/19 (42)
≥6	39/66 (59)	11/19 (58)

Data are presented as No. (%) unless otherwise indicated.

Abbreviations: IQR, interquartile range; PCR^+^, polymerase chain reaction positive.

Lesions were mostly cutaneous (450/511 [88%]): 50% of participants presented skin lesions only, 38% had both skin and mucosal lesions, and 12% had exclusively mucosal lesion(s); 53% of cases had 5 or fewer lesions and 93% had 25 or fewer, in 74% of cases in the perigenital or perianal region. Active skin lesions were mostly vesicles (77%) and pustules (63%). Bacterial superinfection of the lesion occurred in 7% of cases. Lymphadenopathy was found in 48% of participants, fever in 47%, proctitis in 26%, and pharyngitis in 15%. For details, see Table 2. Additional demographic and baseline characteristics can be found in Supplementary Tables 3 and 4. Symptoms in participants with HIV or concomitant STIs were not different from those without (Supplementary Table 5).

**Figure 2.**
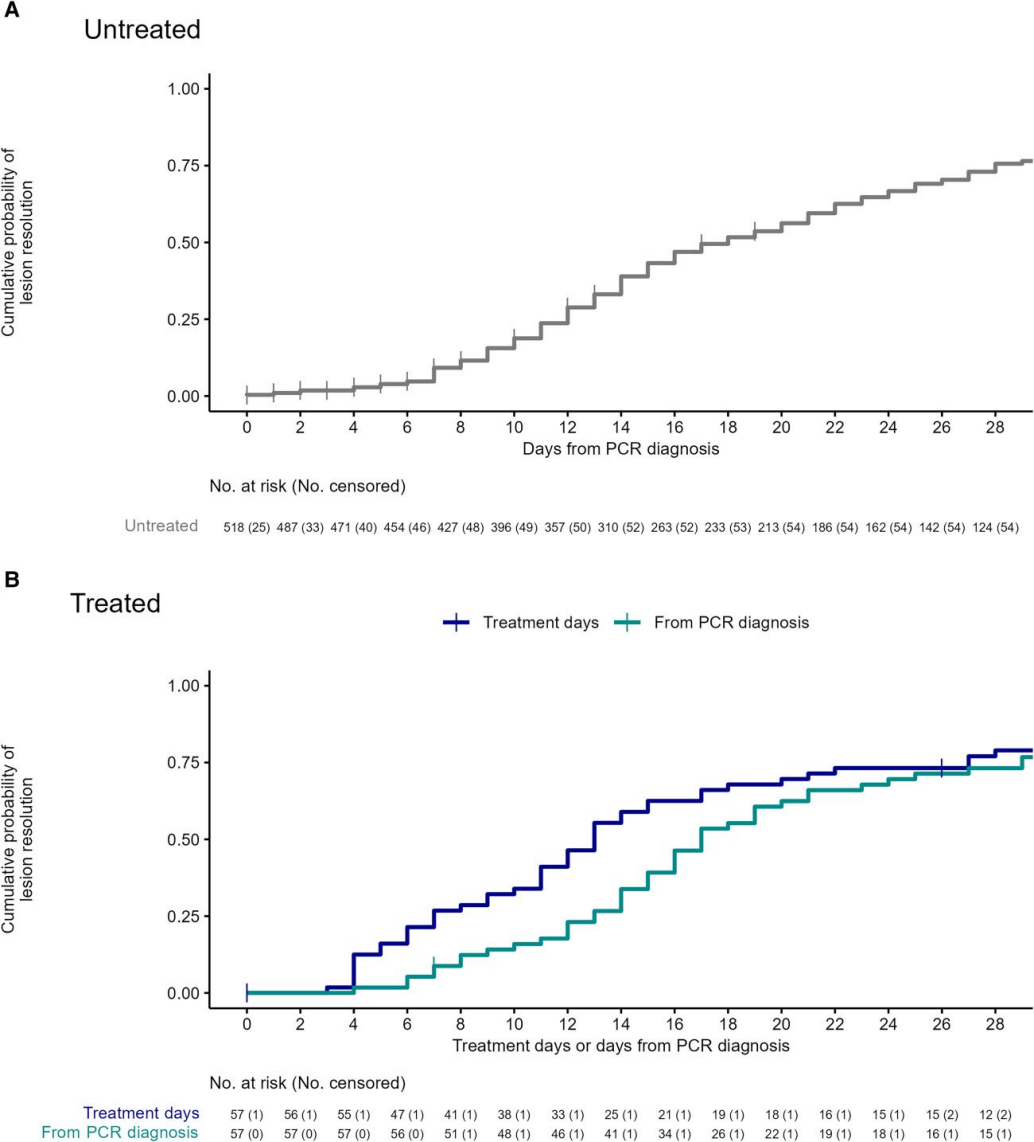
Estimated cumulative probability of lesion resolution based on Kaplan-Meier estimates for untreated participants from the date of polymerase chain reaction (PCR) diagnosis to day 28 (*A*) and treated participants from either date of PCR diagnosis to day 28 or treatment initiation to day 28 (*B*).

**Figure 3.**
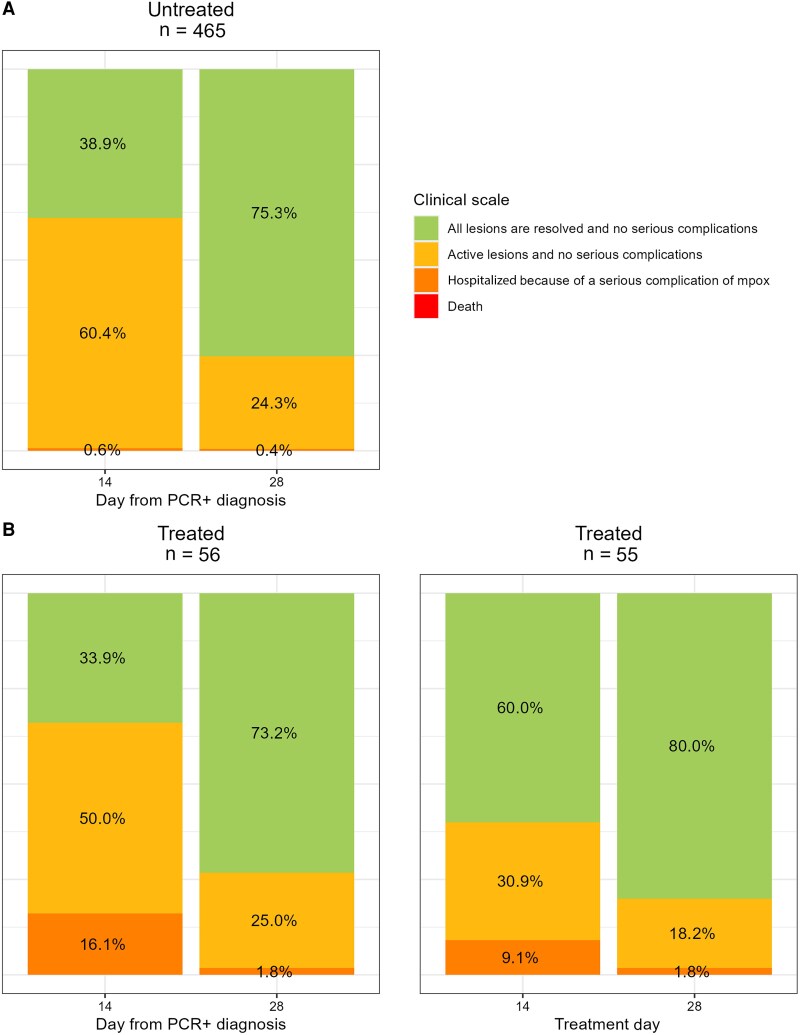
Clinical scales at day 14 and day 28 by treatment status for participants attending the day 14 and day 28 follow-up visits. Abbreviation: PCR, polymerase chain reaction.

**Figure 4.**
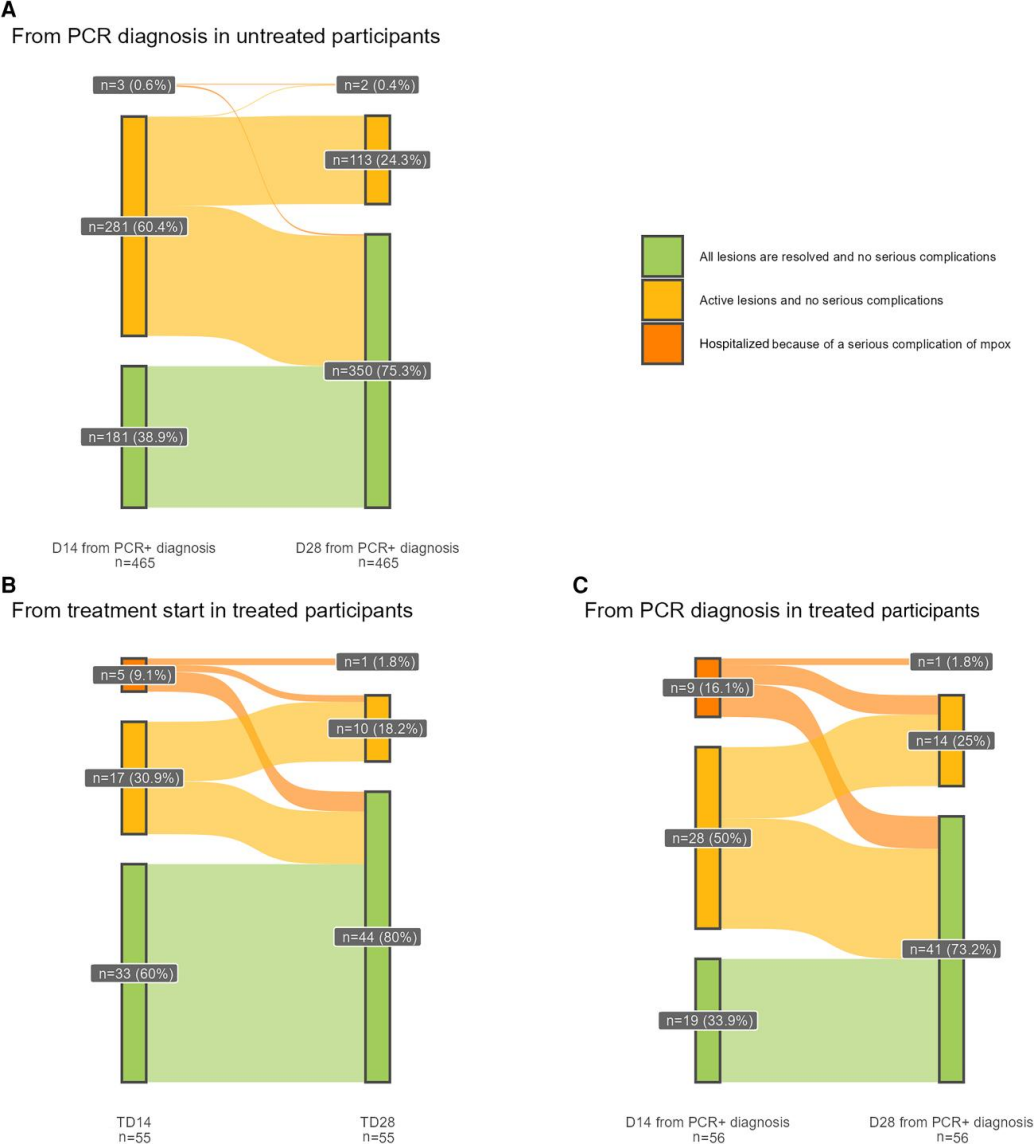
Sankey diagrams from day (D) 14 to D28 for participants attending the D14 and D28 follow-up visits. Abbreviation: PCR, polymerase chain reaction, TD, Treatment Day.

For the primary outcome measure, the KM-estimated proportion of participants with resolved lesions at D14 since the first PCR-positive sample taken was 39% (95% CI, 34%–43%) (Figure 2). The proportion of participants with all lesions resolved and no serious complications increased from 35% on D14 to 68% on D28; 1 and 2 participants, respectively, required hospitalization (Tables 3 and 4; Figures 3 and 4). No evidence of recrudescence or relapse was observed at D60 and D180. No deaths were observed throughout the follow-up period to D28.

**Table 3.** Clinical Outcomes of Participants

Outcome	No. (%)
Untreated	(n = 518)
Day 14	
All lesions are resolved and no serious complications	181 (35)
Active lesions and no serious complications	283 (55)
Hospitalized because of a serious complication of mpox	3 (1)
Death	0 (0)
Loss to follow-up	51 (10)
Day 28	
All lesions are resolved and no serious complications	350 (68)
Active lesions and no serious complications	113 (22)
Hospitalized because of a serious complication of mpox	2 (0)
Death	0 (0)
Loss to follow-up	53 (10)

Treated	From PCR Diagnosis (n = 57)	From Treatment Start (n = 57)
Day 14		
All lesions are resolved and no serious complications	19 (33)	33/57 (58)
Active lesions and no serious complications	28 (49)	18/57 (32)
Hospitalized because of a serious complication of mpox	9 (16)	5/57 (9)
Death	0 (0)	0/57 (0)
Loss to follow-up	1 (2)	1/57 (2)
Day 28		
All lesions are resolved and no serious complications	41 (72)	44/57 (77)
Active lesions and no serious complications	14 (25)	10/57 (18)
Hospitalized because of a serious complication of mpox	1 (2)	1/57 (2)
Death	0 (0)	0/57 (0)
Loss to follow-up	1 (2)	2/57 (4)

Data are presented as No. (%).

Abbreviation: PCR, polymerase chain reaction.

**Table 4.** Summary of Serious Adverse Events per Treatment Group

Adverse Event	Untreated (n = 518)	Treated (n = 57)
Participants with at least 1 SAE	12 (2)	15 (26)
No. of SAEs reported	12	16
List of SAEs (MedDRA SOC and Preferred Terms)		
Infections and infestations	2 (<1)	6 (11)
Abscess	1 (0)	0 (0)
Eye infection intraocular^a^	…	1 (2)
Laryngitis	0 (0)	1 (2)
Pharyngitis	1 (0)	3 (5)
Pharyngotonsillitis	0 (0)	1 (2)
Eye disorders	0 (0)	1 (2)
Ulcerative keratitis	0 (0)	1 (2)
Gastrointestinal disorders	4 (<1)	3 (5)
Proctitis	4 (<1)	3 (5)
General disorders and administration site conditions	5 (1)	1 (2)
Pain	5 (1)	1 (2)
Investigations	0 (0)	1 (2)
Alanine aminotransferase increased	0 (0)	1 (2)
Nervous system disorders	0 (0)	1 (2)
Miller Fisher syndrome	0 (0)	1 (2)
Reproductive system and breast disorders	1 (<1)	1 (2)
Penile edema	1 (<1)	1 (2)
Skin and subcutaneous tissue disorders	0 (0)	2 (4)
Rash^b^	0 (0)	1 (2)
Surgical and medical procedures	0 (0)	1 (2)
Microsurgery to hand	0 (0)	1 (2)

Data are presented as No. (%).

Abbreviations: MedDRA, Medical Dictionary for Regulatory Activities; SAE, serious adverse event; SOC, System Organ Class.

^a^Event described as lesions on the right eyelid with progressive periorbital and conjunctival involvement.

^b^Diffuse rash with de-epithelialization and purulent exudate.

**Figure 5.**
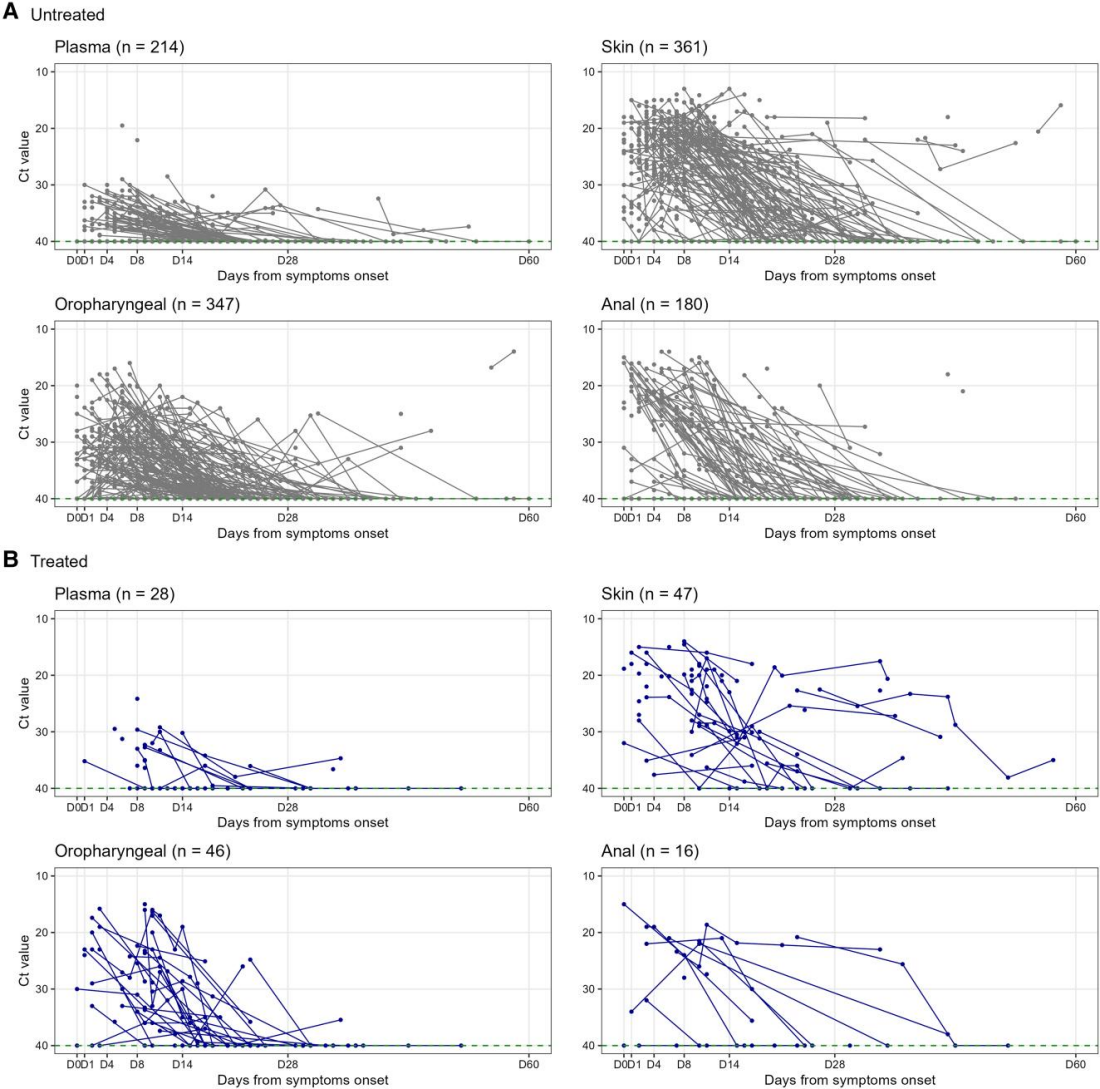
Spaghetti plots of cycle threshold (Ct) values over time for treated and untreated participants with at least 1 virological sample in 1 of the 4 compartments of interest (days [D] since symptom onset).

At least 1 Ct value result was available for 214 participants from a plasma sample, 361 from a skin lesion, 347 from oropharyngeal swab, and 180 from an anal swab (Figure 5). Details on model specifications are in the Supplementary Results. The models for each compartment closely replicated the observed data (see individual fits in Supplementary Figures 1 and 2).

**Figure 6.**
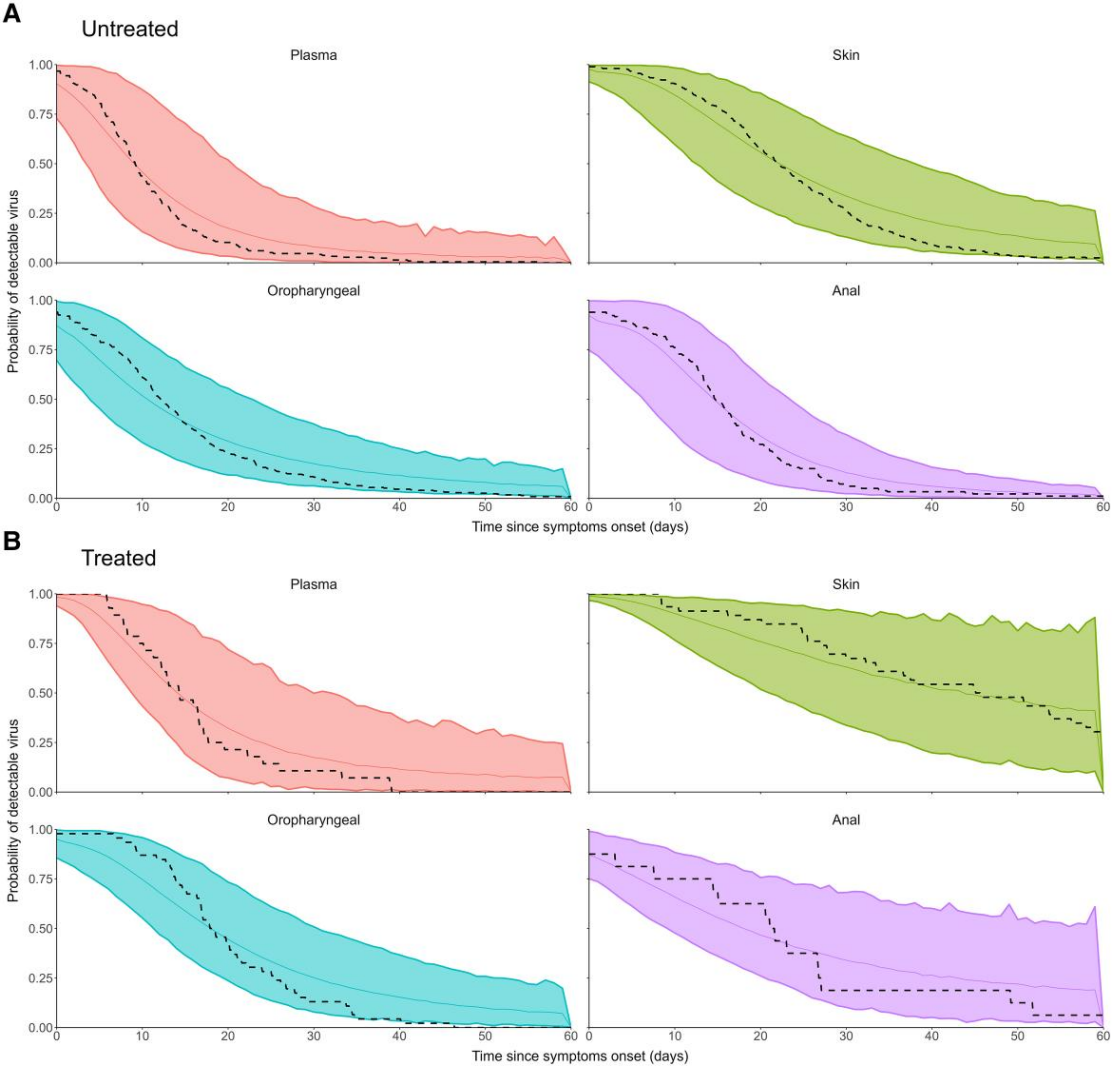
Simulated cumulative incidence of undetectability (mean and 95% confidence intervals [CIs]) for untreated (*A*) and treated (*B*) participants. Means and associated 95% CIs are depicted with solid lines and shaded areas, respectively. The dashed line represents the cumulative incidence calculated on the fitted individuals.

Table 5 presents the simulated predicted times to 50%, 90%, and 95% of participants reaching viral undetectability (Ct ≥40) and nonculturable virus (Ct ≥30), and Figure 6 shows time to undetectability in the different samples. The predicted time to nonculturable virus reached by 95% of participants was 10 days since symptom onset in blood, 30 days in anal, 31 days in oropharyngeal, and 52 days in skin samples.

**Table 5.** Predicted Time to Undetectability (Cycle Threshold [Ct] ≥40) and Predicted Time to Ct ≥30 (From Simulations, in Days)

Sample Location	No.	Predicted Time to Undetectability (Ct ≥40), Mean (95% CI)			Predicted Time to Nonculturable Virus (Ct ≥30), Mean (95% CI)		
		50%	90%	95%	50%	90%	95%
Untreated							
Plasma	214	10 (4–21)	27 (13–57)	39 (17–60)	0 (0–5)	4 (0–36)	10 (0–39)
Skin	361	23 (13–38)	58 (33–60)	60 (42–60)	13 (4–28)	37 (16–60)	52 (22–60)
Oropharyngeal	347	11 (4–23)	44 (23–60)	60 (33–60)	2 (0–11)	19 (6–44)	31 (10–60)
Anal	180	15 (7–24)	34 (20–50)	44 (25–60)	9 (1–17)	23 (11–36)	30 (15–44)
Treated							
Plasma	28	15 (9–30)	44 (19–60)	60 (22–60)	4 (2–7)	13 (7–39)	20 (8–60)
Skin	46	44 (21–60)	60 (58–60)	60 (60–60)	22 (11–60)	60 (32–60)	60 (43–60)
Oropharyngeal	46	18 (12–31)	50 (28–60)	60 (35–60)	9 (4–21)	30 (15–59)	43 (19–60)
Anal	16	19 (10–60)	60 (31–60)	60 (39–60)	8 (4–25)	60 (18–60)	60 (24–60)

Abbreviation: CI, confidence interval; Ct, cycle threshold.

One SAE was reported by 12 participants (2%) between the point of consent and D28 (Table 4), the most frequent being pain at skin or mucosal lesion sites (5 cases) and proctitis (4 cases); there were 5 AEs (Supplementary Table 6).

### Treated Participants

Case management differed across countries: mpox treatment with systemic antivirals ranged from none (Belgium and Spain) to 29% (Switzerland), and hospitalization from none (Belgium) to 36% (Spain) (Supplementary Table 2). Overall, 22 of 57 (39%) required hospitalization for clinical needs, including 2 requiring intensive care (Table 2). A median of 4 days (IQR, 2–8 days) had elapsed from symptom onset to PCR diagnosis and 4 days (IQR, 3–5 days) from diagnosis to treatment start. Treatment was tecovirimat (49 [9%]), cidofovir (6 [1%]), or tecovirimat plus cidofovir (2 [3%]) (Table 6).

**Table 6.** Description of Mpox-Specific Treatments Received Among Treated Participants

Mpox-Specific Treatment	No.
Tecovirimat (± topical treatment)	
Initiated within 14 d of diagnosis	49
Tecovirimat + cidofovir systemic treatment (± topical treatment)	
Tecovirimat and cidofovir initiated within 14 d of diagnosis	1
Cidofovir initiated within 14 d of diagnosis (tecovirimat initiated later)	1
Cidofovir systemic treatment only^a^	
Initiated within 14 d of diagnosis	6

^a^Participants who received cidofovir alone received systemic treatment only; no local treatment was administered.

The demographic and baseline characteristics of the treated cohort generally overlapped with those of untreated participants. As for presenting symptoms, 73% had lymphadenopathy, 56% fever, 33% fever, and 28% pharyngitis/tonsillitis; 33% had >25 lesions with 8% presenting >100. (Table 2).

The KM-estimated proportion of participants with resolved lesions at D14 since the first PCR-positive sample taken was 34% (95% CI, 20%–45%) and 59% (95% CI, 44%–70%) at D14 following treatment initiation. The proportion of participants with resolved lesions with no serious complications increased from 33% on D14 to 72% on D28; 9 (16%) were hospitalized due to a serious complication of mpox infection, and 1 remained hospitalized at D28 (Table 3).

Viral clearance was shortest from blood and longest for skin and anal samples. The predicted time to nonculturable virus by 95% of participants was 20 days in blood, 43 in oropharyngeal samples, and 60 days in skin and anal samples (Table 5).

Fifteen (26%) participants experienced a total of 16 SAEs. The most frequent events were pharyngitis and proctitis (3 each). One SAE was an increase in alanine aminotransferase, which was judged to be possibly treatment related. There were 7 AEs (Supplementary Table 6). Tecovirimat treatment was discontinued for 1 participant following an SAE, a hepatobiliary disorder, and 2 following a liver injury and a hepatic cytolysis AE (Supplementary Table 7).

## DISCUSSION

This article describes clinical presentations and clinical and virologic outcomes of a cohort of mostly adult individuals with laboratory-confirmed clade IIb MPXV infection and active disease, about one-tenth of whom were treated with a systemic antiviral—tecovirimat and/or cidofovir. Compared to previous case series, MOSAIC is the first study to adopt a protocolized approach applied to both prospectively followed participants and retrospectively collected data with a structured case record form, and to have enrolled a large number of cases in the European region. Having in place harmonized, always-on clinical characterization protocols is of immediate relevance, given the new ongoing PHEIC and the complexity of mpox spread involving different clades, variants, and lineages [14].

Presenting signs/symptoms are consistent with other case series; almost 9 in 10 participants had skin lesions, alone or with concomitant mucosal lesions. Half of the participants had mucosal lesions, but mucosal-only lesions were found in only about one-tenth. Lesions were mostly vesicles and/or pustules on presentation and were in three-quarters of cases in the perigenital and perianal areas. Half of the participants had few (≤5) lesions.

HIV status appeared not to influence clinical presentation and outcomes. Participants with HIV had generally normal CD4 cell counts and almost all were on antiretroviral treatment; about half of those who were HIV-negative were on pre- or postexposure prophylaxis. Pre- or postexposure vaccination against smallpox and/or mpox was uncommon.

Cases could mostly be managed on outpatient basis with no specific treatment. Participants treated with a systemic antiviral (predominantly tecovirimat) had proportionally more lesions and almost half of them required hospitalization; this reflects greater propensity to offer treatment to more severe cases, particularly as supplies of antivirals were limited. National treatment guidelines and practices differed and might have been variably followed by individual clinicians (examples are provided in Supplementary Appendix 3), translating into variation across countries in terms of both proportion of cases on systemic antivirals and of cases hospitalized, and complicating a formal comparison of treated versus untreated (Supplementary Table 2).

Viral kinetics are key to understanding disease outcome and transmission potential. We used simulations to calculate the probability of detectable virus (set at Ct ≥40) and of culturable virus (Ct ≥30). Modeled data replicated observed data and are similar to those reported earlier in a smaller cohort from Spain [15], though that study reported viral load in copies per milliliter, while we used Ct and calculated time to culturable virus as a proxy for virus viability and transmission potential. Viral clearance was fastest from plasma and slowest in skin lesions—where it is predicted to take on average about 3 weeks for half of untreated mpox participants to reach viral clearance and 2 weeks to have nonculturable virus from symptom onset. A small proportion of participants (5%) may, however, still carry virus with infectious potential beyond 7 weeks from skin and oropharyngeal lesions. These findings could inform recommendations on reducing risks of onward transmission.

Current options for treating mpox and other orthopoxviruses are limited. Tecovirimat, cidofovir, and its prodrug brincidofovir are variably recommended based on a very incomplete evidence base [16, 17]. Tecovirimat [18] is registered by the EMA [19] and the UK Medicine and Healthcare Products Regulatory Agency for smallpox, mpox, and cowpox as well as vaccinia complications for adults and children weighing ≥13 kg. Since the authorization is based on experimental data and human safety data “under exceptional circumstances” with obligation for “additional monitoring” by the EMA, data generated from this study contribute to safety information of the drug, indicating a potential for altered liver functions. Tecovirimat and brincidofovir are also approved by the US Food and Drug Administration for the treatment of smallpox under the “animal rule” [20].

Despite several interventional and observational studies that are ongoing or planned [21], the evidence base on the effectiveness of mpox treatments remains limited. For the treatment of clade Ia disease, the PALM 007 trial in the Democratic Republic of the Congo found tecovirimat to be safe but not to reduce the duration of mpox lesions over placebo, though the full analyses are not yet available [22]. For clade IIb, available data on tecovirimat in clade IIb mpox are inconclusive and randomized controlled trials are still recruiting at the time of writing [21]. There still remain uncertainties about standardized lesion assessment and disease outcomes that make between-study and between-clade comparisons challenging [23–25].

These results should be seen in the context of study-specific and broader challenges. While the study protocol was developed quickly and enrollment started within 7 weeks from the first cases being reported and before the WHO declared the mpox outbreak a PHEIC [26], complex regulations significantly slowed down the study setup in several participating countries under the current European Clinical Trials Regulation, which could only begin enrolling participants when cases were dwindling. In a related article, we describe these shortcomings and propose improvements [27]. MOSAIC offers important lessons for the clinical research community in terms of observational data collection and helped the International Severe Acute Respiratory and Emerging Infection Consortium adapt its standardized tools for the rapid and harmonized collection of data and biological samples [28] that are made freely available and can now be applied to the new ongoing PHEIC.

## Supplementary Material

ciae657_Supplementary_Data

## Supplementary Data

Supplementary materials are available at *Clinical Infectious Diseases* online. Consisting of data provided by the authors to benefit the reader, the posted materials are not copyedited and are the sole responsibility of the authors, so questions or comments should be addressed to the corresponding author.

## Notes

***Acknowledgments.*** Members of the MOSAIC Study Group are listed in the Appendix below.

***Financial support*.** This work was supported by the UK Foreign, Commonwealth and Development Office and Wellcome (grant number 215091/Z/18/Z) and the Bill & Melinda Gates Foundation (grant number OPP1209135). In the European Union, additional funding is provided by MPX-Response funded by the European Commission under the HE program (101115188).

## APPENDIX

**Participating sites and investigators:**

Maya Hites (Hôpital Universitaire de Bruxelles-Érasme, Université Libre de Bruxelles [ULB], Belgium), Leïla Belkhir (UCL St Luc, Bruxelles, Belgium), Marie-Angélique De Scheerder (UZ Gent), Jean-Christophe Goffard (Cliniques Universitaires de Bruxelles Hôpital Érasme, ULB, Brussels, Belgium), Agnès Libois (Department of Infectious Diseases, Centre hospitalier universitaire [CHU] Saint-Pierre, ULB, Brussels, Belgium), Zineb Khalil (Cliniques Universitaires de Bruxelles Hôpital Érasme, ULB, Brussels, Belgium), Catherine Orban (CHU Liège, Belgium), Lucie Seyler (Department of Infectious Diseases, Érasme Hospital, ULB, Brussels, Belgium), Clotilde Visée (Pôle Hospitalier Jolimont/site de Mons-Warquignies), Florence Ader (Département des Maladies Infectieuses et Tropicales, Hospices Civils de Lyon, France), Antoine Bachelard (Assistance Publique–Hôpitaux de Paris **[**AP-HP] Nord, Hôpital Bichat, Department of Epidemiology Biostatistics and Clinical Research, Paris, France), Yasmine Bouaraba (AP-HP Nord, Hôpital Bichat, Department of Epidemiology Biostatistics and Clinical Research, Paris, France), Jean-Marc Chapplain (CHU Pontchaillou), Hugues Cordel (Hôpital Avicenne), François Danion (Department of Infectious Diseases, CHU de Strasbourg, France), Aurélien Dinh (Hôpital Raymond Poincaré), Nikita Dobremel (AP-HP Nord, Hôpital Bichat, Department of Epidemiology Biostatistics and Clinical Research, Paris, France), Manuel Etienne (Hôpital Charles Nicolle, Rouen, France), Pierre Frange (AP-HP Centre, Hôpital Necker Enfants Malades, Paris, France), Karine Faure (Hôpital Claude Huriez, Lille, France), François Goehringer (Centre hospitalier régional universitaire de Nancy, France), Karine Lacombe (Hôpital Saint Antoine, Paris, France), Xavier Lescure (AP-HP Nord, Hôpital Bichat, Department of Epidemiology Biostatistics and Clinical Research, Paris, France), Rodolphe Manaquin (CHU de La Réunion, St Pierre, France), Guillaume Martin-Blondel (CHU Toulouse, France), Jean-Michel Mansuy (CHU Toulouse, France), Giovanny Gombert (CHU Toulouse, France), Duc Nguyen (Hôpital Pellegrin, Bordeaux, France), Romain Palich (Sorbonne University, Infectious Diseases Department, Pitié-Salpêtrière hospital, AP-HP, Pierre-Louis Epidemiology and Public Health Institute [iPLESP], Institut national de la santé et de la recherche médicale [INSERM] U1136, Paris, France), Gilles Pialoux (Hôpital Tenon, Paris, France), Aoife Cotter (Mater Misericordiae University Hospital Dublin, Ireland), Virginie Gautier (Infectious Diseases Department, University College Dublin, Ireland), Evelina Tacconelli (University of Verona, Italy), Oriana Awwad (University of Verona, Italy; University of Jordan, Amman), Andrea Antinori (National Institute for Infectious Diseases Lazzaro Spallanzani, IRCCS, Rome, Italy), Antonella Castagna (Vita-Salute University, IRCCS San Raffaele Scientific Institute, Milan, Italy), Silvia Nozza (Vita-Salute University, IRCCS San Raffaele Scientific Institute, Milan, Italy), Angelo Roberto Raccagni (Vita-Salute San Raffaele University, Milan, Italy), Elena Bruzzesi (Vita-Salute San Raffaele University, Milan, Italy), Antonio Cascio (University of Palermo, Italy), Annamaria Cattelan (Azienda Ospedale Università Padova, Italy), Maddalena Cordioli (University of Verona, Italy), Giulia De Luca (University of Verona, Italy), Lorenza Lambertenghi (University of Verona, Italy), Giulia Marchetti (San Paolo Hospital, Milan, Italy), Valentina Mazzotta (National Institute for Infectious Diseases Lazzaro Spallanzani, IRCCS, Rome, Italy), Emanuele Nicastri (National Institute for Infectious Diseases Lazzaro Spallanzani, IRCCS, Rome, Italy), Vincenzo Scaglione (Azienda Ospedale Università Padova, Italy), Carlo Tascini (Udine University Hospital, Italy), Jorge Machado (Instituto Nacional de Saudé, Lisbon, Portugal), Lurdes Santos (Infectious Diseases Service, ID Intensive Care Unit, Faculdade de Medicina, Centro Hospitalar Universitário de São João, Universidade do Porto, Portugal), Wong Chen Seong (National Centre for Infectious Diseases, Singapore), Travis Ren Teen Chia (National Centre for Infectious Diseases, Singapore), Wilnard Yeong Tze Tan (National Centre for Infectious Diseases, Singapore), Fernando De La Calle Prieto (National Reference Centre for Imported Pathology and International Health High-Level Isolation Unit, La Paz-Carlos III Hospital, IdiPAZ, CIBERINFEC, Madrid, Spain), Vicente Estrada (Hospital Clínico Universitario Madrid, Spain), Miguel Nicolás Navarrete Lorite (Hospital Virgen de la Macarena, Sevilla, Spain), Alfredo Soler Carracedo (Hospital Universitario La Paz, Madrid, Spain), Maria Velasco Arribas (Hospital Fundación Alcorcón, Madrid, Spain), Dominique Braun (Department of Infectious Diseases and Hospital Epidemiology, University Hospital Zurich, Switzerland), Alexandra Calmy (HIV/AIDS Unit, Division of Infectious Diseases, Geneva University Hospitals, Switzerland), Matthias Cavassini (Service of Infectious Diseases, CHU Vaudois, Lausanne, Switzerland), Lorenzo Ciullini (Maladies infectieuses, Hôpital du Valais, Sion, Switzerland), Stéphane Emonet (Maladies infectieuses, Hôpital du Valais, Sion, Switzerland), Chiara Fedeli (HIV/AIDS Unit, Division of Infectious Diseases, Geneva University Hospitals, Switzerland), Yvan Gosmain (HIV/AIDS Unit, Division of Infectious Diseases, Geneva University Hospitals, Switzerland), Laetitia Guiraud (HIV/AIDS Unit, Division of Infectious Diseases, Geneva University Hospitals, Switzerland), Benjamin Hampel (Checkpoint Zurich, Zurich, Switzerland), Olivier Segeral (Geneva University Hospitals, Switzerland), Cornelia Staehelin (Department of Infectious Diseases, Inselspital, Bern University Hospital, University of Bern, Switzerland), Marcel Stöckle (Division of Infectious Diseases and Hospital Epidemiology, University Hospital Basel, University of Basel, Switzerland), Alejandro Arenas-Pinto (Central and North West London NHS Foundation Trust, London, UK), Mike Beadsworth (Liverpool University Hospitals NHS Foundation Trust, Liverpool, UK), Margherita Bracchi (Chelsea & Westminster Hospitals NHS Foundation Trust, London, UK), Joby Cole (Sheffield Teaching Hospitals NHS Foundation Trust, Sheffield, UK), Jake Dunning (Royal Free London NHS Foundation Trust, London, UK), Michael Marks (University College London Hospitals NHS Foundation Trust, London, UK), Brendan Payne (Newcastle Hospitals NHS Foundation Trust, Royal Victoria, Newcastle, UK), Malcolm G. Semple (National Institute for Health and Care Research, Health Protection Research Unit in Emerging and Zoonotic Infections, Institute of Infection, Veterinary and Ecological Sciences, Faculty of Health and Life Sciences, University of Liverpool, UK), Claire Waddington (Imperial College Healthcare NHS Trust, London, UK), Chris Ward (St George's University Hospitals NHS Foundation Trust, London, UK)

**Statistical advisers:**

Alain Amstutz (Division of Clinical Epidemiology, Department of Clinical Research, University Hospital Basel and University of Basel, Switzerland; Oslo Center for Biostatistics and Epidemiology, Oslo University Hospital, Norway; Population Health Sciences, Bristol Medical School, University of Bristol, UK), Dominique Costagliola (Sorbonne Université, INSERM, Institut Pierre-Louis d’Épidémiologie et de Santé Publique, Paris, France), Jérémie Guedj (INSERM, France), Inge Christoffer Olsen (Department of Research Support for Clinical Trials, Oslo University Hospital, Norway)

**Virology task force:**

Diane Descamps (AP-HP Nord, Hôpital Bichat, Department of Virology, Université Paris Cité, INSERM UMR 1137, Paris, France), Christophe Batejat (Cellule d’intervention biologique d’urgence [CIBU], Institut Pasteur, France), Jessica Vanhomwegen (CIBU, Institut Pasteur, France), Maude Bouscambert-Duchamp (Department of Virology, Hospices Civils de Lyon, France), Julio Garcia Rodriguez (Virology, Hospital la Paz, Spain), Alexandre Gaymard (Department of Virology, Hospices Civils de Lyon, France), Andreas Lind (Microbiology Department, Oslo University, Norway), Fabrizio Maggi (Laboratory of Virology, National Institute for Infectious Diseases Lazzaro Spallanzani, IRCCS, Rome, Italy), Hervé Raoul (INSERM–National Agency for Research on AIDS and Viral Hepatitis [ANRS] Emerging Infectious Diseases [MIE], France), Jeroen Van Kampen (Viroscience Department, Netherlands), Sabine Yerly (Geneva University Hospitals, Switzerland), Nicolas Yin (Department of Microbiology, Laboratoire Hospitalier Universitaire de Bruxelles, ULB, Brussels, Belgium)

**French methodological and management center and sponsor representative:**

Léo Chenard (Hôpital Bichat, Paris, France), Ismaila Deme (Hôpital Bichat, Paris, France), Alpha Diallo (INSERM–ANRS MIE, France), Séverine Gibowski (INSERM–ANRS MIE, France), Sabrina Kali (INSERM–ANRS MIE, France), Guillaume Le Meut (INSERM–ANRS MIE, France), Sophie Letrou (Hôpital Bichat, Paris, France), Claire Madelaine (INSERM–ANRS MIE, France), Hervé Raoul (INSERM–ANRS MIE, France), Ventzislava Petrov Sanchez (INSERM–ANRS MIE, France).

## References

[1] Petersen E, Kantele A, Koopmans M, et al Human monkeypox: epidemiologic and clinical characteristics, diagnosis, and prevention. Infect Dis Clin North Am 2019; 33:1027–43. 30981594 10.1016/j.idc.2019.03.001 PMC9533922

[2] Cho W, Park S, Kim HJ, et al Clinical characteristics and outcomes of patients with mpox during the 2022 mpox outbreak compared with those before the outbreak: a systematic review and meta-analysis. Rev Med Virol 2024; 34:e2508. 38282393 10.1002/rmv.2508

[3] Thornhill JP, Barkati S, Walmsley S, et al Monkeypox virus infection in humans across 16 countries—April–June 2022. N Engl J Med 2022; 387:679–91. 35866746 10.1056/NEJMoa2207323

[4] Patel A, Bilinska J, Tam JCH, et al Clinical features and novel presentations of human monkeypox in a central London centre during the 2022 outbreak: descriptive case series. BMJ 2022; 378:e072410. 35902115 10.1136/bmj-2022-072410 PMC9331915

[5] Tarín-Vicente EJ, Alemany A, Agud-Dios M, et al Clinical presentation and virological assessment of confirmed human monkeypox virus cases in Spain: a prospective observational cohort study. Lancet 2022; 400:661–9. 35952705 10.1016/S0140-6736(22)01436-2 PMC9533900

[6] Antinori A, Mazzotta V, Vita S, et al Epidemiological, clinical and virological characteristics of four cases of monkeypox support transmission through sexual contact, Italy, May 2022. Euro Surveill 2022; 27:2200421. 35656836 10.2807/1560-7917.ES.2022.27.22.2200421 PMC9164671

[7] Mailhe M, Beaumont AL, Thy M, et al Clinical characteristics of ambulatory and hospitalized patients with monkeypox virus infection: an observational cohort study. Clin Microbiol Infect 2023; 29:233–9. 36028090 10.1016/j.cmi.2022.08.012 PMC9533921

[8] Fink DL, Callaby H, Luintel A, et al Clinical features and management of individuals admitted to hospital with monkeypox and associated complications across the UK: a retrospective cohort study. Lancet Infect Dis 2023; 23:589–97. 36566771 10.1016/S1473-3099(22)00806-4

[9] Pesonel E, Hoffmann I, Guiraud L, et al MOSAIC: a cohort study of human mpox virus disease. Wellcome Open Res 2023; 8:415. 38031544 10.12688/wellcomeopenres.19616.3 PMC10685065

[10] Kali S, Bourner J, Calmy A, et al MOSAIC: a European cohort study of human mpox—the challenges of clinical research in outbreaks. Virologie (Montrouge) 2023; 27:12–5. 36896770 10.1684/vir.2023.986

[11] Harris PA, Taylor R, Thielke R, Payne J, Gonzalez N, Conde JG. Research electronic data capture (REDCap)—a metadata-driven methodology and workflow process for providing translational research informatics support. J Biomed Inform 2009; 42:377–81. 18929686 10.1016/j.jbi.2008.08.010 PMC2700030

[12] Harris PA, Taylor R, Minor BL, et al The REDCap consortium: building an international community of software platform partners. J Biomed Inform 2019; 95:103208. 31078660 10.1016/j.jbi.2019.103208 PMC7254481

[13] Brown EG, Wood L, Wood S. The Medical Dictionary for Regulatory Activities (MedDRA). Drug Saf 1999; 20:109–17. 10082069 10.2165/00002018-199920020-00002

[14] World Health Organization . WHO Director-General declares mpox outbreak a Public Health Emergency of International Concern. 2024. Available at: https://www.who.int/news/item/14-08-2024-who-director-general-declares-mpox-outbreak-a-public-health-emergency-of-international-concern. Accessed 11 September 2024.

[15] Suñer C, Ubals M, Tarín-Vicente EJ, et al Viral dynamics in patients with monkeypox infection: a prospective cohort study in Spain. Lancet Infect Dis 2023; 23:445–53. 36521505 10.1016/S1473-3099(22)00794-0 PMC9977560

[16] Delaune D, Iseni F. Drug development against smallpox: present and future. Antimicrob Agents Chemother 2020; 64:e01683-19. 31932370 10.1128/AAC.01683-19 PMC7179270

[17] Wang J, Shahed-Ai-Mahmud M, Chen A, Li K, Tan H, Joyce R. An overview of antivirals against monkeypox virus and other orthopoxviruses. J Med Chem 2023; 66:4468–90. 36961984 10.1021/acs.jmedchem.3c00069

[18] Sherwat A, Brooks JT, Birnkrant D, Kim P. Tecovirimat and the treatment of monkeypox—past, present, and future considerations. N Engl J Med 2022; 387:579–81. 35921403 10.1056/NEJMp2210125

[19] European Medicines Agency . Tecovirimat SIGA. 2023. Available at: https://www.ema.europa.eu/en/medicines/human/EPAR/tecovirimat-siga. Accessed 25 July 2024.

[20] US Food and Drug Administration . FDA mpox response. 2022. Available at: https://www.fda.gov/emergency-preparedness-and-response/mcm-issues/fda-mpox-response#:∼:text=TPOXX%20(tecovirimat)%20and%20Tembexa%20(,humans%2C%20for%20example%2C%20in%20the. Accessed 25 July 2024.

[21] Olliaro P, Bourner J, Boum Ii Y, et al Mpox: the alarm went off. Have we gone back to sleep? PLoS Negl Trop Dis 2024; 18:e0011871. 38236842 10.1371/journal.pntd.0011871 PMC10796058

[22] National Institute of Allergy and Infectious Diseases . The antiviral tecovirimat is safe but did not improve clade I mpox resolution in Democratic Republic of the Congo. 2024. Available at: https://www.niaid.nih.gov/news-events/antiviral-tecovirimat-safe-did-not-improve-clade-i-mpox-resolution-democratic-republic. Accessed 11 September 2024.

[23] Jones B, Paterson A, AlKhoury N, et al Variability in clinical assessment of clade IIb mpox lesions. Int J Infect Dis 2023; 137:60–2. 37848125 10.1016/j.ijid.2023.10.004 PMC10914632

[24] Bourner J, Garcia-Gallo E, Mbrenga F, et al Challenges in clinical diagnosis of clade I mpox: highlighting the need for enhanced diagnostic approaches. PLoS Negl Trop Dis 2024; 18:e0012087. 38913721 10.1371/journal.pntd.0012087 PMC11226010

[25] Rojek A, Dunning J, Olliaro P. Monkeypox: how will we know if the treatments work? Lancet Infect Dis 2022; 22:1269–70. 35931096 10.1016/S1473-3099(22)00514-X PMC9629677

[26] World Health Organization . WHO Director-General declares the ongoing monkeypox outbreak a Public Health Emergency of International Concern. 2022. Available at: https://www.who.int/europe/news/item/23-07-2022-who-director-general-declares-the-ongoing-monkeypox-outbreak-a-public-health-event-of-international-concern. Accessed 11 September 2024.

[27] Patrick-Brown T, Bourner J, Kali S, et al Experiences and challenges with the new European Clinical Trials Regulation. Trials 2024; 25:3. 38167484 10.1186/s13063-023-07869-x PMC10759753

[28] ISARIC mpox research response guidance. 2024. Available at: https://isaricresearch.github.io/Responses/Mpox. Accessed 11 September 2024.

