# Supplementary material for: Clinical Characterization and Outcomes of Human Clade IIb Mpox Virus Disease: A European Multicenter Mpox Observational Cohort Study (MOSAIC)
Source: Clin Infect Dis. 2025 Jan 3;80(5):1060–73. doi: 10.1093/cid/ciae657 (PMC12135908; doi:10.1093/cid/ciae657)
Supplement: ciae657_Supplementary_Data [file ciae657_supplementary_data.docx]

**Supplementary Material**

Supplement to Clinical characterisation and outcomes of human clade IIb mpox virus disease – a European multicentre observational cohort study (MOSAIC).

**Contents**

[**Supplementary Methods** 2](#_Toc184920718)

[**Statistical model for Ct values** 2](#_Toc184920719)

[**Supplementary Results** 3](#_Toc184920720)

[**Table S1 – Enrolment by country** 3](#_Toc184920721)

[**Table S2 – Participants hospitalised at enrolment by country and treatment status** 4](#_Toc184920722)

[**Table S3 – All participant characteristics reported at baseline** 5](#_Toc184920723)

[**Table S4 – Other participant characteristics reported at baseline of all, treated and untreated mpox participants** 6](#_Toc184920724)

[**Table S5 – Participant characteristics reported at baseline of all, HIV+, HIV- participants, participants with other STI and no other STI.** 1](#_Toc184920725)

[**Table S6 – List of Adverse Events (AEs) per treatment group** 1](#_Toc184920726)

[**Table S7 – Summary of actions taken following SAE or AE in treated participants** 2](#_Toc184920727)

[**Supplementary results on virology modelling** 3](#_Toc184920728)

[**Model specifications** 3](#_Toc184920729)

[**Figure S1 – Individual fits of 30 untreated participants** 3](#_Toc184920730)

[**Figure S2 – Individual fits of 30 treated participants** 4](#_Toc184920731)

[**Supplementary Appendix** 5](#_Toc184920732)

[**Appendix 1 – Data Dictionary** 5](#_Toc184920733)

[**Appendix 2 – Summary of responsible ethics committees and respective reference numbers** 20](#_Toc184920734)

[**Appendix 3 – MPXV recommendations and practices in MOSAIC participants Countries** 21](#_Toc184920735)

# **Supplementary Methods**

## **Statistical model for Ct values**

The model used is as follows:

$$y_{ij}^{k}=f\left( t_{ij}, \psi_{i}, k \right)+ e_{ij}^{k} (1)$$

where $y_{ij}^{k}$ is the $j^{th}$ observation of subject $i$ at time $t_{ij}$ of compartment $k$, with $i\in1,\ldots, N$, the number of subjects, and $j\in1,\ldots n_{i}$, the number of observations of subject $i$ and $k$ the compartment. $f\left( t_{ij}, \psi_{i}, k \right)$ describes the Ct value at time $t_{ij}$ of subject $i$ in compartment $k$, with a linear structure, and is written as follows: patie

$$f\left( t_{ij}, \psi_{i}, k \right)=\beta_{0ik}+\beta_{1ik}t_{ij} (2)$$

$\beta_{0}$ is the hypothetical baseline Ct value at symptom onset and $\beta_{1}$ is the increase in Ct values per day. The vector of individual parameters $\psi_{i}$ depends on a fixed effect vector and an individual random effect vector, which follows a normal centered distribution with diagonal variance-covariance matrix $\Omega$. We assumed a lognormal distribution for both $\beta_{0}$ and $\beta_{1}$ parameters to ensure their positivity. The residual gaussian error $e_{ij}^{k}$ is of constant standard deviation $\sigma$. Correlation between random effects were added and kept only if they were above 0.7 and did not degrade the relative standard errors (RSEs) of population parameters to above 50%. The time between symptom onset and diagnosis (in days) was tested as a continuous covariate on both the intercept and slope, respectively $\beta_{0}$ and $\beta_{1}$, and kept in the model only if significant and did not degrade the RSEs of population parameters to above 50%. We assumed all compartments to be independent and fitted them separately. The limit of detection was set at 40 Ct in all compartments, and values above this limit were considered right censored. Parameters were estimated using the stochastic approximation expectation maximisation algorithm implemented in Monolix 2024 R1^^[[1]](#footnote-2)^^.

**Simulations to compute the probabilities of detectable and culturable virus**

We sampled 500 population parameters in their asymptotic estimation distribution and then, for each set of population parameters, we sampled 300 individual parameters (resulting in 150,000 individual parameters per compartment). We then calculated the time to virus undetectability at both Ct thresholds for each individual in each dataset, and the predicted probability of detectable and culturable virus in each dataset as mean and 95% confidence interval over all datasets at all times^[[2]](#footnote-3)^.

# **Supplementary Results**

## **Table S1 – Enrolment by country**

|  | **Participants enrolled** | **Eligible participants** |
| --- | --- | --- |
| **TOTAL** | **619** | **602** |
| **BELGIUM** | **72** | **72** |
| Hôpital Erasme -Bruxelles | 6 | 6 |
| Cliniques universitaires Saint-Luc | 3 | 3 |
| CHU Saint-Pierre Site Porte de Hal | 63 | 63 |
| **SWITZERLAND** | **43** | **39** |
| Hôpitaux Universitaires de Genève | 13 | 13 |
| Universitätsspital Basel | 4 | 3 |
| CHUV Centre Hospitalier Universitaire Vaudois | 10 | 8 |
| Universitätsspital Zürich | 7 | 6 |
| Zürich Checkpoint | 8 | 8 |
| Hôpital du Valais | 1 | 1 |
| **SPAIN** | **16** | **16** |
| Hospital Universitario La Paz | 6 | 6 |
| Hospital Fundación Alcorcón | 10 | 10 |
| **FRANCE** | **180** | **167** |
| Hôpital Tenon AP-HP | 12 | 12 |
| Hôpitaux Universitaires Pitié Salpêtrière | 45 | 43 |
| Hôpital Saint-Antoine AP-HP | 8 | 8 |
| CHU de Bordeaux | 2 | 2 |
| Hôpital Bichat | 75 | 69 |
| Centre Hospitalier Universitaire de Toulouse | 6 | 6 |
| CHRU de Nancy | 1 | 0 |
| Centre Hospitalier Universitaire de Rouen | 4 | 3 |
| Hospices Civils de Lyon | 12 | 12 |
| Centre Hospitalier Universitaire de La Réunion | 3 | 3 |
| Hôpitaux Universitaires de Strasbourg | 6 | 4 |
| Centre Hospitalier Universitaire de Lille | 6 | 5 |
| **ITALY** | **273** | **273** |
| University of Verona | 10 | 10 |
| National Institute for Infectious Diseases Lazzaro Spallanzani | 80 | 80 |
| San Raffaele Scientific Institute and Hospital | 138 | 138 |
| University of Palermo | 9 | 9 |
| University Hospital of Udine | 2 | 2 |
| Azienda Ospedale Università Padova | 23 | 23 |
| San Paolo Hospital | 11 | 11 |
| **UNITED KINGDOM** | **35** | **35** |
| Imperial College Healthcare NHS Trust | 10 | 10 |
| Liverpool University Hospitals NHSFT | 2 | 2 |
| Royal Free London NHSFT | 5 | 5 |
| Sheffield Teaching Hospital | 4 | 4 |
| Central and North West London NHSFT | 10 | 10 |
| Chelsea and Westminster NHSFT | 3 | 3 |
| University College London Hospitals | 1 | 1 |

## **Table S2 – Participants hospitalised at enrolment by country and treatment status**

| **Country** | **Analysed total** | **Hospitalised total (%)** | **Treated total** | **Treated hospitalised (%)** | **Untreated total** | **Unreated hospitalised (%)** |
| --- | --- | --- | --- | --- | --- | --- |
| Belgium | 71 | 0 (0) | 0 | 0 (0) | 71 | 0 (0) |
| Spain | 14 | 5 (35.7) | 0 | 0 (0) | 14 | 5 (35.7) |
| France | 162 | 16 (9.9) | 8 | 6 (75) | 154 | 10 (6.5) |
| Italy | 258 | 17 (6.6) | 24 | 5 (20.8) | 234 | 12 (5.1) |
| Switzerland | 36 | 6 (16.7) | 15 | 3 (20) | 21 | 3 (14.3) |
| United Kingdom | 34 | 12 (35.3) | 10 | 6 (60) | 24 | 6 (25) |
| **Total** | **575** | **56 (89.7)** | **57** | **20 (35.1)** | **518** | **36 (6.9)** |

## **Table S3 – All participant characteristics reported at baseline**

|  |  | | **All participants (N=575)** | |
| --- | --- | --- | --- | --- |
| Demographics |  | |  | |
| Age - Median (IQR) - yr |  | | 37 [32 ; 44] | |
| Female sex - no/total no (%) |  | | 6/572 (1) | |
| Concomitant active sexually transmitted infection |  | | 113/570 (21) | |
| Syphilis |  | | 53/113 (47) | |
| Mycoplasma genitalium |  | | 9/113 (8) | |
| Chlamydia |  | | 19/113 (17) | |
| Hepatitis B |  | | 3/113 (3) | |
| HSV |  | | 7/113 (6) | |
| Gonorrhoea |  | | 33/113 (29) | |
| HPV |  | | 2/113 (2) | |
| Ureaplasma |  | | 6/113 (5) | |
| Hepatitis C |  | | 5/570 (1) | |
| HIV/ AIDS + |  | | 239/574 (42) | |
| Receiving ARV |  | | 228/239 (95) | |
| Viral load detectable |  | | 30/216 (14) | |
| Viral load - Median (IQR) - cp/ml |  | | 708 [56 ; 96117] | |
| CD4-T at last follow up - Median (IQR) /µl |  | | 731 [522 ; 1034] | |
| Receiving HIV PREP / PEP |  | | 165/335 (49) | |
|  |  |  | |  |
| Management after clinical presentation - no/total no (%) | | |  | |
| Outpatient | | | 497/571 (87) | |
| Admitted to hospital ward for isolation only | | | 15/571 (3) | |
| Admitted to hospital ward for clinical need | | | 56/571 (10) | |
| Intensive care unit admission | | | 3/571 (1) | |
| Time from - Median (IQR), days | | |  | |
| Symptom onset to PCR+ diagnosis | | | 5 [3 ; 8] | |
| PCR+ diagnosis to enrolment | | | 0 [0 ; 0] | |
| PCR+ diagnosis to treatment start | | | - | |
| Symptoms - no/total no (%) | | |  | |
| Lymphadenopathy | | | 283/561 (50) | |
| Fever | | | 269/562 (48) | |
| Headache | | | 121/565 (21) | |
| Pharyngitis/tonsillitis | | | 92/567 (16) | |
| Lower respiratory symptoms | | | 11/567 (2) | |
| Ocular complications | | | 10/567 (2) | |
| Diarrhoea/gastroenteritis | | | 41/567 (7) | |
| Nausea/Vomiting | | | 14/566 (2) | |
| Encephalitis | | | 2/566 (0.4) | |
| Other complications  Proctitis - no/total no (%) | | | 142/566 (25) | |
| Bacterial super-infection - no/total no (%) | | | 29/345 (8) | |
| Folliculitis/cellulitis | | | 14/29 (48) | |
| Bacteraemia | | | 1/29 (3) | |
| Urinary tract | | | 1/29 (3) | |
| Other | | | 13/29 (45) | |
| Mpox lesions: | | |  | |
| Skin / mucosal lesions - no/total no (%) | | |  | |
| Skin lesions only | | | 280/567 (49) | |
| Mucosal lesions only | | | 66/567 (12) | |
| Skin and mucosal lesions | | | 221/567 (39) | |
| Active skin lesions types - no/total no (%) | | |  | |
| Vesicle | | | 257/330 (78) | |
| Pustule | | | 216/331 (65) | |
| Ulcerated lesion | | | 91/331 (27) | |
| Haemorrhagic / bleeding lesions | | | 5/257 (2) | |
| Other | | | 25/341 (7) | |
| Lesion sites - no/total no (%) | | |  | |
| Head skin lesions | | | 204/567 (36) | |
| Ocular lesions within the orbit | | | 2/567 (0) | |
| Other skin lesions | | | 303/568 (53) | |
| Mucosal lesions | | | 287/567 (51) | |
| Peri-genital or peri-anal lesions | | | 422/567 (74) | |
| Estimated total number of active lesions- no/total no (%) | | |  | |
| 1-5 | | | 247/492 (50) | |
| 6-25 | | | 199/492 (40) | |
| 26-100 | | | 39/492 (8) | |
| >100 | | | 7/492 (1) | |
| Pain score - Median (IQR) - out of 10 | | | 6 [3 ; 8] | |
| Dichotomized pain score - no/total no (%) | | |  | |
| pain score <6 | | | 35/85 (41) | |
| pain score >=6 | | | 50/85 (59) | |

## **Table S4 – Other participant characteristics reported at baseline of all, treated and untreated mpox participants**

|  |  | **All participants (N=575)** | **Treated (N=57)** | **Untreated (N=518)** |
| --- | --- | --- | --- | --- |
| **Demographics** |  |  |  |  |
| Ethnicity - no/total no (%) |  |  |  |  |
| White |  | 426/554 (77) | 41/57 (72) | 385/497 (77) |
| Latin American |  | 49/554 (9) | 4/57 (7) | 45/497 (9) |
| Black |  | 41/554 (7) | 5/57 (9) | 36/497 (7) |
| Arab |  | 24/554 (4) | 2/57 (4) | 22/497 (4) |
| West Asian |  | 3/554 (1) | 1/57 (2) | 2/497 (0) |
| South Asian |  | 2/554 (0) | 1/57 (2) | 1/497 (0) |
| Other |  | 9/554 (2) | 3/57 (5) | 6/497 (1) |
| **Comorbidities** - no/total no (%) |  |  |  |  |
| Chronic cardiac disease |  | 4/574 (1) | 2/57 (4) | 2/517 (0) |
| Chronic respiratory disease (e.g., asthma, COPD) |  | 8/574 (1) | 1/57 (2) | 7/517 (1) |
| Chronic kidney disease |  | 3/574 (1) | 1/57 (2) | 2/517 (0) |
| Moderate / severe liver disease |  | 11/574 (2) | 2/57 (4) | 9/517 (2) |
| Diabetes mellitus and type |  |  |  |  |
| Yes - type 1 |  | 2/574 (0) | 1/57 (2) | 1/517 (0) |
| Yes - type 2 |  | 1/574 (0) | 0/57 (0) | 1/517 (0) |
| Chronic neurological disorder |  | 4/573 (1) | 1/57 (2) | 3/516 (1) |
| Malignant neoplasm |  | 0/572 (0) | 0/56 (0) | 0/516 (0) |
| Chronic hematologic disease |  | 1/572 (0) | 0/56 (0) | 1/516 (0) |
| **Immunosuppressant Treatments** - no/total no (%) |  |  |  |  |
| Corticosteroid use |  | 5/571 (1) | 2/57 (4) | 3/514 (1) |
| Other immunosuppressant use |  | 5/572 (1) | 2/57 (4) | 3/515 (1) |
| **Smallpox/Mpox vaccination** |  |  |  |  |
| Yes - Pre-exposure indication |  | 38/526 (7) | 2/53 (4) | 36/473 (8) |
| Yes - Post-exposure indication |  | 13/526 (2) | 1/53 (2) | 12/473 (3) |
| Scar but not confirmed |  | 35/526 (7) | 1/53 (2) | 34/473 (7) |

## **Table S5 – Participant characteristics reported at baseline of all, HIV+, HIV- participants, participants with other STI and no other STI.**

|  | **All participants** | **HIV+** | **HIV-** | **Other STI +** | **No other STI** |
| --- | --- | --- | --- | --- | --- |
|  | **(N=575)** | **(N=239)** | **(N=335)** | **(N=113)** | **(N=457)** |
| **Management after clinical presentation** - no/total no (%) |  |  |  |  |  |
| Outpatient | 497/571 (87) | 208/237 (88) | 288/333 (86) | 97/110 (88) | 396/456 (87) |
| Admitted to hospital ward for isolation only | 15/571 (3) | 1/237 (0) | 14/333 (4) | 0/110 (0) | 15/456 (3) |
| Admitted to hospital ward for clinical need | 56/571 (10) | 27/237 (11) | 29/333 (9) | 13/110 (12) | 42/456 (9) |
| Intensive care unit admission | 3/571 (1) | 1/237 (0) | 2/333 (1) | 0/110 (0) | 3/456 (1) |
| **Symptoms** - no/total no (%) |  |  |  |  |  |
| Pharyngitis/tonsillitis | 92/108 (85) | 34/37 (92) | 58/71 (82) | 14/17 (82) | 76/88 (86) |
| Lymphadenopathy | 283/561 (50) | 113/232 (49) | 169/328 (52) | 48/107 (45) | 232/450 (52) |
| Fever | 269/562 (48) | 108/233 (46) | 161/328 (49) | 45/108 (42) | 221/449 (49) |
| Headache | 121/565 (21) | 46/233 (20) | 75/331 (23) | 17/108 (16) | 100/452 (22) |
| Lower respiratory symptoms | 11/108 (10) | 3/37 (8) | 8/71 (11) | 2/17 (12) | 8/88 (9) |
| Ocular complications | 10/108 (9) | 2/37 (5) | 8/71 (11) | 1/17 (6) | 8/88 (9) |
| Diarrhoea/gastroenteritis | 41/567 (7) | 20/235 (9) | 21/331 (6) | 7/108 (6) | 33/454 (7) |
| Nausea/Vomiting | 14/566 (2) | 5/234 (2) | 9/331 (3) | 2/108 (2) | 12/453 (3) |
| Encephalitis | 2/107 (2) | 0/37 (0) | 2/70 (3) | 0/17 (0) | 2/88 (2) |
| **Other complications** | 142/566 (25) | 76/233 (33) |  | 39/108 (36) |  |
| **Proctitis** - no/total no (%) |  |  | 66/332 (20) |  | 102/453 (23) |
| **Bacterial super-infection** - no/total no (%) | 29/345 (8) | 11/132 (8) | 18/212 (8) | 4/57 (7) | 25/286 (9) |
| Folliculitis/cellulitis | 14/29 (48) | 8/11 (73) | 6/18 (33) | 3/4 (75) | 11/25 (44) |
| Bacteraemia | 1/29 (3) | 1/11 (9) | 0/18 (0) | 0/4 (0) | 1/25 (4) |
| Urinary tract | 1/29 (3) | 0/11 (0) | 1/18 (6) | 0/4 (0) | 1/25 (4) |
| Other | 13/29 (45) | 2/11 (18) | 11/18 (61) | 1/4 (25) | 12/25 (48) |
| **Mpox lesions:** |  |  |  |  |  |
| **Skin / mucosal lesions** - no/total no (%) |  |  |  |  |  |
| Skin lesions only | 280/567 (49) | 108/236 (54) | 171/330 (48) | 47/108 (56) | 231/454 (49) |
| Mucosal lesions only | 66/567 (12) | 33/236 (14) | 33/330 (10) | 18/108 (17) | 48/454 (11) |
| Skin and mucosal lesions | 221/567 (39) | 95/236 (40) | 126/330 (38) | 43/108 (40) | 175/454 (39) |
| **Active skin lesions types** - no/total no (%) |  |  |  |  |  |
| Vesicle | 257/330 (78) | 114/140 (81) | 143/189 (76) | 50/67 (75) | 204/260 (78) |
| Pustule | 216/331 (65) | 89/140 (64) | 127/190 (67) | 46/67 (69) | 168/261 (64) |
| Ulcerated lesion | 91/331 (27) | 32/140 (23) | 58/190 (31) | 16/67 (24) | 75/262 (29) |
| Haemorrhagic / bleeding lesions | 5/257 (2) | 2/102 (2) | 3/154 (2) | 0/51 (0) | 5/203 (2) |
| Other | 25/341 (7) | 7/144 (5) | 18/196 (9) | 2/69 (3) | 22/269 (8) |
| **Lesion sites** - no/total no (%) |  |  |  |  |  |
| Head skin lesions | 204/567 (36) | 94/236 (40) | 110/330 (33) | 38/108 (35) | 165/454 (36) |
| Ocular lesions within the orbit | 2/567 (0) | 1/236 (0) | 1/330 (0) | 0/108 (0) | 2/454 (0) |
| Other skin lesions | 303/568 (53) | 120/236 (51) | 183/331 (55) | 54/108 (50) | 246/455 (54) |
| Mucosal lesions | 287/567 (51) | 128/236 (54) | 159/330 (48) | 61/108 (56) | 223/454 (49) |
| Peri-genital or peri-anal lesions | 422/567 (74) | 179/236 (76) | 242/330 (73) | 84/108 (78) | 333/454 (73) |
| **Estimated total number of active lesions**- no/total no (%) |  |  |  |  |  |
| 1-5 | 247/492 (50) | 98/198 (49) | 148/293 (51) | 46/95 (48) | 198/393 (50) |
| 6-25 | 199/492 (40) | 82/198 (41) | 117/293 (40) | 42/95 (44) | 156/393 (40) |
| 26-100 | 39/492 (8) | 16/198 (8) | 23/293 (8) | 7/95 (7) | 32/393 (8) |
| >100 | 7/492 (1) | 2/198 (1) | 5/293 (2) | 0/95 (0) | 7/393 (2) |

## **Table S6 – List of Adverse Events (AEs) per treatment group**

| **MedDRA SOC**  **MedDRA Preferred Term** | **Treated**  **(N=57)** | **Untreated (N=518)** |
| --- | --- | --- |
| N (%) participants with at least 1 AE | 7 (12%) | 5 (1%) |
| N AEs reported | 9 | 5 |
| List of SAEs (**MedDRA SOC** and **Preferred Terms)** |  |  |
| **Infections and infestations** | **2 (4%)** | **2 (<1%)** |
| Cytomegalovirus infection | 1 (2%) | 0 (0%) |
| Giardiasis | 0 (0%) | 1 (0%) |
| Skin infection | 1 (2%) | 0 (0%) |
| Superinfection | 0 (0%) | 1 (0%) |
| **General disorders and administration site conditions** | **0 (0%)** | **1 (<1%)** |
| Influenza like illness | 0 (0%) | 1 (0%) |
| **Hepatobilary disorders** | **2 (4%)** | **0 (0%)** |
| Hepatic cytolysis | 1 (2%) | 0 (0%) |
| Liver injury | 1 (2%) | 0 (0%) |
| **Investigations** | **2 (4%)** | **0 (0%)** |
| Alanine aminotransferase increased | 1 (2%) | 0 (0%) |
| SARS-CoV-2 test positive | 1 (2%) | 0 (0%) |
| **Musculoskeletal and connective tissue disorders** | **1 (2%)** | **1 (<1%)** |
| Myalgia | 0 (0%) | 1 (<1%) |
| Tendonitis | 1 (2%) | 0 (0%) |
| **Metabolism and nutrition disorders** | **1 (2%)** | **0 (0%)** |
| Starvation | 1 (2%) | 0 (0%) |
| **Renal and urinary disorders** | **1 (2%)** | **1 (<1%)** |
| Dysuria | 0 (0%) | 1 (<1%) |
| Urinary retention | 1 (2%) | 0 (0%) |

| **SAE Event**  **MedDRA Preferred Term** | **Mpox treatment received** | **Actions taken**  **following SAE** | **Relationship of the event to mpox treatment** |
| --- | --- | --- | --- |
| Alanine aminotransferase increased | TECOVIRI  MAT | Permanently discontinued | Possibly related |
| Laryngitis | CIDOFOVIR | No action taken | Not related |
| Microsurgery to hand | TECOVIRIMAT | No action taken | Not related |
| Miller Fisher syndrome | TECOVIRIMAT | No action taken | Not related |
| Mpox | CIDOFOVIR | No action taken | Not related |
| Pain | CIDOFOVIR | No action taken | Not related |
| Penile oedema | TECOVIRIMAT | No action taken | Not related |
| Pharyngitis | TECOVIRIMAT | No action taken | Not related |
| Pharyngitis | TECOVIRIMAT | No action taken | Not related |
| Pharyngitis | CIDOFOVIR | No action taken | Not related |
| Pharyngotonsillitis | TECOVIRIMAT | No action taken | Not related |
| Proctitis | TECOVIRIMAT | No action taken | Not related |
| Proctitis | TECOVIRIMAT | No action taken | Not related |
| Proctitis | TECOVIRIMAT | No action taken | Not related |
| Rash | TECOVIRIMAT | No action taken | Not related |
| Ulcerative keratitis | CIDOFOVIR | No action taken | Not related |
| **AE Event**  **MedDRA Preferred Term** | **Mpox treatment received** | **Actions taken**  **following AE** |  |
| Alanine aminotransferase increased | TECOVIRIMAT | No action taken |  |
| Cytomegalovirus infection | TECOVIRIMAT | No action taken |  |
| Hepatic cytolysis | TECOVIRIMAT | Permanently discontinued |  |
| Liver injury | TECOVIRIMAT | Permanently discontinued |  |
| SARS-CoV-2 test positive | TECOVIRIMAT | No action taken |  |
| Skin infection | TECOVIRIMAT | No action taken |  |
| Starvation | TECOVIRIMAT | No action taken |  |
| Tendonitis | TECOVIRIMAT | No action taken |  |
| Urinary retention | TECOVIRIMAT | Not known |  |

## **Table S7 – Summary of actions taken following SAE or AE in treated participants**

# **Supplementary results on virology modelling**

## **Model specifications**

The additive error term was not estimated and fixed to 3 Ct in all models.

In the untreated group, the model for each compartment incorporated the time between symptom onset and diagnosis as a covariate on the intercept, and no correlation between the random effects was added in any of the four models. For treated participants, no covariate and no correlation between random effects were added for the plasma, skin and anal compartments because it resulted in degraded relative standard errors for the population parameters; the time between symptom onset and diagnosis was incorporated as a covariate on the intercept in the oropharyngeal compartment model.

## **Figure S1 – Individual fits of 30 untreated participants**

Solid lines represent model prediction. Empty dots are data above the limit of detection (i.e. Ct ≥ 40).


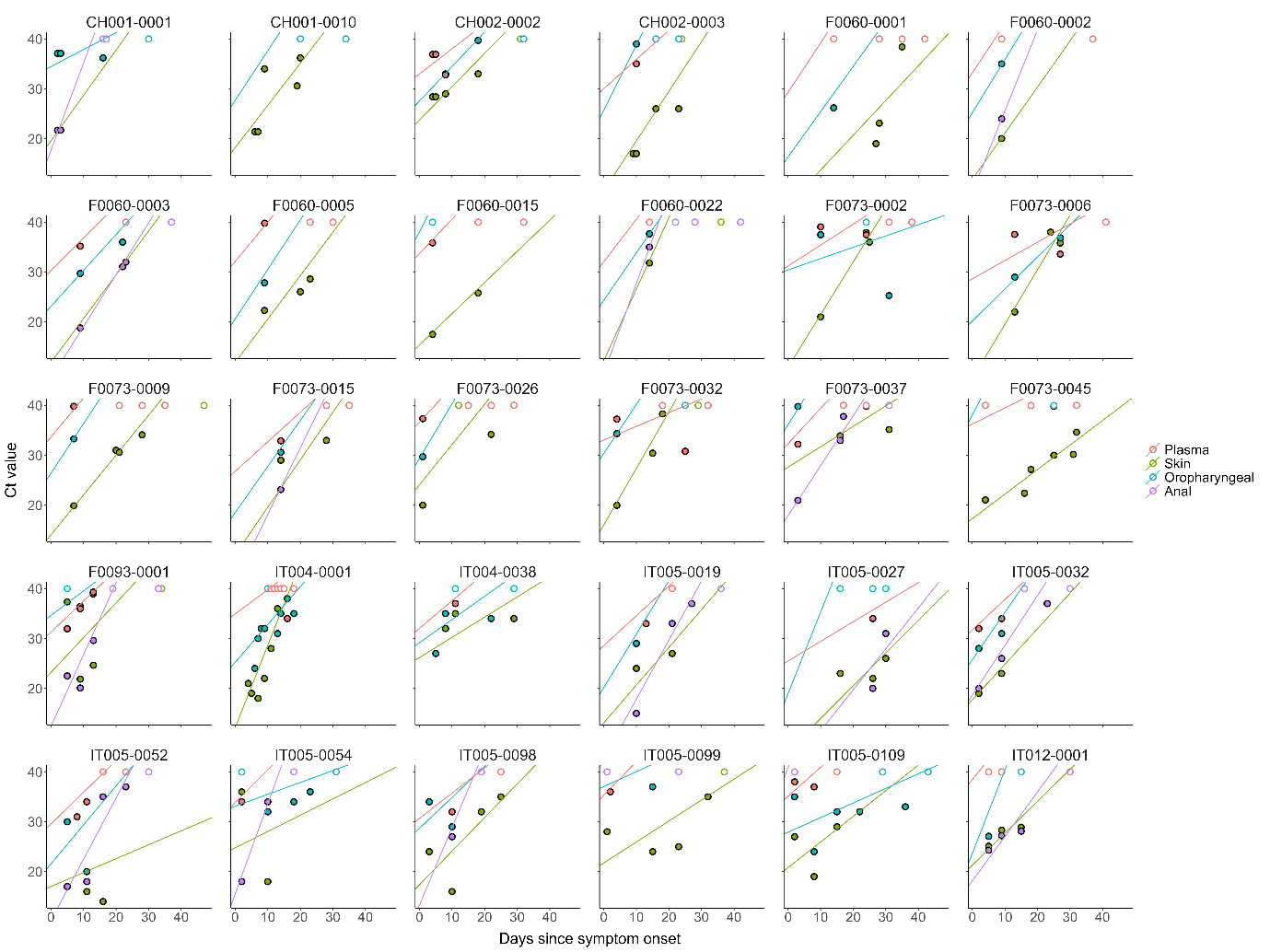


## **Figure S2 – Individual fits of 30 treated participants**

Solid lines represent model prediction. Empty dots are data above the limit of detection (i.e. Ct ≥ 40).


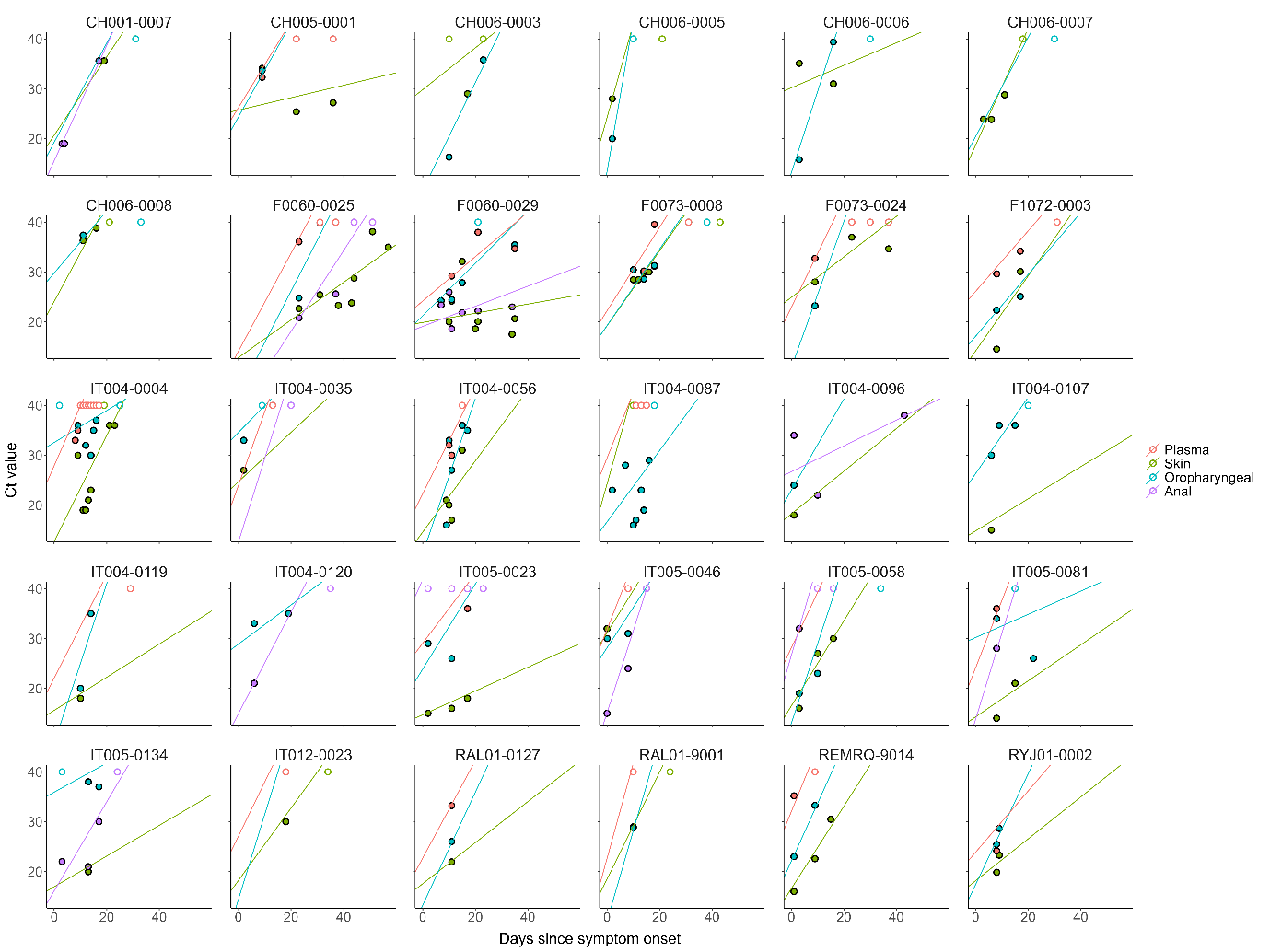


# **Supplementary Appendix**

## **Appendix 1 – Data Dictionary**

| **Section Header** | **Variable** | **Choices** |
| --- | --- | --- |
| **BASELINE ASSESSMENT** |  |  |
| **ENROLMENT** | Date of evaluation |  |
|  | Systemic symptoms | 1, Yes \| 0, No |
|  | Date of onset of systemic symptoms |  |
|  | Skin lesions | 1, Yes \| 0, No |
|  | Date of onset of skin lesions |  |
|  | Genital lesions | 1, Yes \| 0, No |
|  | Date of onset of genital lesions |  |
|  | Mucosal lesions | 1, Yes \| 0, No |
|  | Date of onset of mucosal lesions |  |
|  | Type of mucosal lesions: | 1, Anal/perianal \| 2, Oral |
|  | Date of first symptom onset |  |
|  | Date of contact with infected person |  |
|  | Type of exposure | 1, Community contact \| 2, Vertical transmission \| 3, Healthcare worker contact |
|  | Before the current episode of monkeypox, has the participant ever been infected with the monkeypox virus? |  |
|  | When did the previous episode of mpox begin? |  |
|  | Approximately when did the previous episode of monkeypox end? |  |
|  | Was the episode of monkeypox confirmed by laboratory testing (e.g. testing of a monkeypox lesion swab, or rectal swab?) |  |
|  | Has the participant been enrolled in MOSAIC prior to recruitment for their current episode of monkeypox? |  |
|  | If yes, please indicate the participant's previous ID: |  |
| **ELIGIBILITY CRITERIA** | Does the patient have PCR confirmation of monkeypox infection? | 1, Yes \| 0, No (exclude) \| 2, Sample pending |
|  | PLEASE COMPLETE THE WITHDRAWAL FORM |  |
|  | Date PCR sample taken |  |
|  | Consent |  |
|  | Has the patient (or their legal representative) provided written informed consent to be included in the study? | 1, Yes \| 0, No (exclude) |
|  | France only - Has the patient (or their legal representative) had no opposition to be included in the study? |  |
|  | UK only Was consent obtained by a nominated consultee? | 1, Yes \| 0, No |
|  | Samples cannot be collected for this patient |  |
|  | Does the participant agree for their anonymised samples to be used in future mpox research ? |  |
|  | Date of consent |  |
|  | Please do not record any patient into REDCap without a date of consent</center> |  |
|  | Name of the person taking consent |  |
|  | Type of consent (e.g. Adults, Children ....) |  |
|  | Version date of the consent |  |
|  | Version number of the consent |  |
| **DEMOGRAPHICS** | Sex at Birth | 1, Male \| 2, Female \| 3, Not specified |
|  | Is date of birth known? |  |
|  | Date of birth |  |
|  | If date of birth is Not Known (N/K) record Age: |  |
|  | Age units, years or months? | 2, Years \| 1, Months |
|  | Weight in kg |  |
|  | Ethnic group (check all that apply): | 1, Arab \| 2, Black \| 3, East Asian \| 4, South Asian \| 11, South East Asian \| 5, West Asian \| 6, Latin American \| 7, White \| 8, Aboriginal/First Nations \| 9, Other |
|  | Specify other ethnic group |  |
|  | Pregnant? | 1, Yes \| 0, No |
|  | If pregnant: Gestational age |  |
|  | Breastfeeding? |  |
| **COMORBIDITIES & TREATMENTS** | Chronic cardiac disease, including congenital heart disease (not hypertension). | 1, Yes \| 0, No |
|  | Chronic respiratory disease (e.g., asthma, COPD) | 1, Yes \| 0, No |
|  | Chronic kidney disease | 1, Yes \| 0, No |
|  | Moderate / severe liver disease | 1, Yes \| 0, No |
|  | Diabetes mellitus and type | 1, Yes - type 1 \| 2, Yes - type 2 \| 0, No |
|  | Chronic neurological disorder | 1, Yes \| 0, No |
|  | Malignant neoplasm | 1, Yes \| 0, No |
|  | Chronic hematologic disease | 1, Yes \| 0, No |
|  | Active sexually transmitted infection (e.g., HepB, Syphilis) | 1, Yes \| 0, No |
|  | Specify active sexually transmitted infection |  |
|  | HIV / AIDS | 1, Yes - on ARV \| 2, Yes - not treated \| 0, No |
|  | HIV viral load | 0, Not detectable \| 1, Detectable |
|  | HIV viral load |  |
|  | CD4-T at last follow up: per microliter |  |
|  | On HIV PREP / PEP | 1, Yes \| 0, No |
|  | Corticosteroid use | 1, Yes \| 0, No |
|  | CD4-T at last follow up: % |  |
|  | Other immunosuppressant use | 1, Yes \| 0, No |
|  | Specify other immunosuppressant name and indication |  |
| **SMALLPOX VACCINATION** | Did the patient receive smallpox vaccination? (including vaccine during childhood) | 1, Yes - Pre-exposure prophylaxis \| 2, Yes - Post-exposure prophylaxis \| 3, Scar but not confirmed \| 0, No \| 5, N/K |
|  | Name of vaccine |  |
|  | How many doses did the patient receive? | 1, 1 Dose \| 2, 2 Doses \| 9, N/K |
|  | Is date of last vaccination dose known? |  |
|  | Date of last dose of vaccination |  |
|  | If date is approximately known, please record here: |  |
| **MONKEYPOX TREATMENT** | Was the patient treated before, or is the patient treated after enrolment date? | 1, Yes \| 0, No \| 2, Unknown |
|  | Tecovirimat | 1, Yes \| 0, No |
|  | Date of tecovirimat start |  |
|  | Duration of prescribed treatment (days) |  |
|  | Duration of the treatment taken (in days) (if known): |  |
|  | Was the duration of treatment extended? |  |
|  | If yes, by how many days? |  |
|  | Brincidofovir | 1, Yes \| 0, No |
|  | Date of brincidofovir start |  |
|  | Duration of prescribed treatment (days) |  |
|  | Duration of the treatment taken (in days) (if known): |  |
|  | Was the duration of treatment extended? |  |
|  | If yes, by how many days? |  |
|  | Cidofovir | 1, Yes \| 0, No |
|  | Date of cidofovir start |  |
|  | Duration of prescribed treatment (days) |  |
|  | Duration of the treatment taken (in days) (if known): |  |
|  | Was the duration of treatment extended? |  |
|  | If yes, by how many days? |  |
|  | Use of Cidofovir treatment | 1, Systemic \| 2, Local |
|  | Why it was used for mpox or other reasons |  |
|  | Was Cidofovir used as it was the only available treatment? | 1, Yes \| 0, No |
|  | Other | 1, Yes \| 0, No |
|  | Name of other treatment |  |
|  | Date of treatment start |  |
|  | Duration of prescribed treatment (days) |  |
|  | Duration of the treatment taken (in days) (if known): |  |
|  | Was the duration of treatment extended? |  |
|  | If yes, by how many days? |  |
|  | Date of assessment |  |
| **DAILY ASSESSSMENT (everyday for inpatients; baseline, day 14, day 28, month 2, month 6 for all)** |  |  |
|  | Current level of care | 1, Outpatient \| 2, Admitted to normal ward for isolation only \| 3, Admitted to normal ward for clinical care \| 4, Intensive care admission |
|  | Fever | 1, Yes \| 0, No |
|  | Temperature (C) |  |
|  | Lymphadenopathy | 1, Yes \| 0, No |
|  | Headache | 1, Yes \| 0, No |
|  | Nausea/Vomiting | 1, Yes \| 0, No |
|  | Diarrhoea/gastroenteritis | 1, Yes \| 0, No |
|  | Encephalitis | 1, Yes \| 0, No |
|  | Ocular complications | 1, Yes \| 0, No |
|  | Pharyngitis/tonsillitis | 1, Yes \| 0, No |
|  | Lower respiratory symptoms (productive cough, wheezing, respiratory distress) | 1, Yes \| 0, No |
|  | Specify lower respiratory symptoms |  |
|  | Deep tissue abscess | 1, Yes \| 0, No |
|  | Ano-rectitis | 1, Yes \| 0, No |
|  | New sexually transmitted infection (post baseline) | 1, Yes \| 0, No |
|  | Bacterial super-infection | 1, Yes \| 0, No |
|  | Bacterial super-infection | 1, Folliculitis/cellulitis \| 2, Pneumonia \| 3, Gastroenteritis \| 4, CNS \| 5, Bacteraemia \| 6, Urinary tract \| 7, Other |
|  | Please specify other bacterial infection |  |
|  | Do you think there has been an adverse reaction to a monkeypox treatment in the last 24 hours? | 1, Yes \| 0, No |
|  | PLEASE COMPLETE AN ADVERSE EVENT FORM |  |
| **DAILY LESION ASSESSMENT** | Please note that the lesion assessment is only for lesions due to Mpox |  |
|  | SKIN LESIONS<div> |  |
|  | Which is true of skin lesions today | 1, There are active skin lesions \| 2, There are skin lesions, and none of them are active \| 0, All skin lesions have completely resolved (all scabs have fallen off with intact skin) \| 3, No skin lesions have developed to date |
|  | Are the following types of active skin lesions currently present? (Select at least one yes) |  |
|  | Vesicle (active) | 1, Yes \| 0, No |
|  | Pustule (active) | 1, Yes \| 0, No |
|  | Ulcerated lesion (active) | 1, Yes \| 0, No |
|  | Haemorrhagic / bleeding lesions (active) | 1, Yes \| 0, No |
|  | Other active lesion type | 1, Yes \| 0, No |
|  | Residual evidence of resolved lesions (scar/discoloration - inactive) | 1, Yes \| 0, No |
|  | MUCOSAL LESIONS |  |
|  | Please note that any mucosal lesion is considered an active lesion. |  |
|  | Which is true of mucosal lesions today | 1, There are mucosal lesions \| 0, There are no mucosal lesions, any mucosal lesions have completely resolved (mucosa is healed) \| 2, No mucosal lesions have developed to date |
|  | Persistant symptoms of rectitis | 1, Yes \| 2, No |
|  | Anal or rectal pain | 1, Yes \| 0, No |
|  | Rectal bleeding or discharge | 1, Yes \| 0, No |
|  | Sensation of needing to pass stool when bowels are empty (tenesmus) | 1, Yes \| 0, No |
|  | Persistent symptoms or signs of mucosal oropharyngeal lesions | 1, Yes \| 0, No |
|  | Pharyngitis, angina | 1, Yes \| 0, No |
|  | Oro-pharyngeal ulcerated lesion (active) | 1, Yes \| 0, No |
|  | SKIN & MUCOSAL LESIONS |  |
|  | Are there active skin or mucosal lesions in the following areas? (Select at least one yes) |  |
|  | Head, face, neck | 1, Yes \| 0, No |
|  | Inside of mouth | 1, Yes \| 0, No |
|  | Torso | 1, Yes \| 0, No |
|  | Arms and/or hands | 1, Yes \| 0, No |
|  | Legs and/or feet | 1, Yes \| 0, No |
|  | External genitalia | 1, Yes \| 0, No |
|  | Perianal | 1, Yes \| 0, No |
|  | Vaginal canal | 1, Yes \| 0, No |
|  | Rectum | 1, Yes \| 0, No |
|  | Other | 1, Yes \| 0, No |
|  | Specify other lesion location |  |
|  | Have new skin or mucosal lesions appeared in the previous 24 hours? (patients may be best placed to assess this) | 1, Yes \| 0, No |
|  | Estimate total number of active skin or mucosal lesions on the body: | 1, None \| 2, 1-5 \| 3, 6-25 \| 4, 26-100 \| 5, >100 \| 6, N/K |
|  | Pain at any skin or mucosal lesion site? | 1, Yes \| 0, No |
|  | Maximal pain score out of 10, where 0 means no pain and 10 means the worst possible pain. |  |
|  | Describe any other lesion complications: |  |
|  | Is this assessment post day 14 (days 28, 60, 180 or extra visit)? | 1, Yes \| 0, No |
| **FOR VISITS POST DAY 14** | Has the patient experienced any new spots or lesions after the first episode of lesions resolved (first episode = when recruited to the study)? | 1, Yes \| 0, No |
| *(Day 28, Day 60, Day 180, Extra visit)* | Has data on this secondary episode been collected during a previous visit (please check previous follow-up visit data)? If yes, please verify completeness and accuracy of the previously recorded data. Do not repeat the data here. |  |
|  | On what date did the patient first notice any lesion of the second episode? |  |
|  | On what date did the last lesion of the second episode appear? |  |
|  | Does the patient feel that all lesions have healed? | 1, Yes \| 0, No |
|  | On what date does the patient feel that all lesions had healed? |  |
|  | If not yet healed, please review on next visit and update these data |  |
|  | How many skin or mucosal lesions occurred during this second episode (so far, if ongoing)? | 1, None \| 2, 1-5 \| 3, 6-25 \| 4, 26-100 \| 5, >100 \| 6, N/K |
| **On what parts of the body did the lesion(s) occur during the second episode?** | Head, face, neck | 1, Yes \| 0, No |
|  | Inside of mouth | 1, Yes \| 0, No |
|  | Torso | 1, Yes \| 0, No |
|  | Arms and/or hands | 1, Yes \| 0, No |
|  | Legs and/or feet | 1, Yes \| 0, No |
|  | External genitalia | 1, Yes \| 0, No |
|  | Perianal | 1, Yes \| 0, No |
|  | Vaginal canal | 1, Yes \| 0, No |
|  | Rectum | 1, Yes \| 0, No |
|  | Other | 1, Yes \| 0, No |
|  | Specify other lesion location | 1, Yes 0, No |
| **Check all symptoms that the patient had AT ANY POINT during the second episode of lesions.** | Fever | 1, Yes \| 0, No |
|  | Lymphadenopathy | 1, Yes \| 0, No |
|  | Headache | 1, Yes \| 0, No |
|  | Nausea/vomiting | 1, Yes \| 0, No |
|  | Diarrhoea/gastroenteritis | 1, Yes \| 0, No |
|  | Encephalitis | 1, Yes \| 0, No |
|  | Ocular complications | 1, Yes \| 0, No |
|  | Pharyngitis/tonsillitis | 1, Yes \| 0, No |
|  | Lower respiratory symptoms (productive cough, wheezing, respiratory distress) | 1, Yes \| 0, No |
|  | Deep tissue abscess | 1, Yes \| 0, No |
|  | Ano-rectitis | 1, Yes \| 0, No |
|  | Did the patient seek medical attention during the second episode of lesions? | 1, Yes \| 0, No |
|  | Did the clinician say they had mpox? | 1, Yes \| 0, No |
|  | Did they have a swab taken for mpox? | 1, Yes \| 0, No |
|  | Record the result in an Additional labs form |  |
| **MONKEYPOX TREATMENT** | Is the patient receiving tecovirimat treatment? | 1, Yes \| 0, No |
|  | Please keep the "MPOX treatment" section in the Baseline Assessment Form up to date |  |
|  | For how many days has the patient received tecovirimat? | 1, 1 (Treatment Day 1, started today) \| 2, 2 (started yesterday) \| 3, 3 \| 4, 4 \| 5, 5 \| 6, 6 \| 7, 7 \| 8, 8 \| 9, 9 \| 10, 10 \| 11, 11 \| 12, 12 \| 13, 13 \| 14, 14 \| 15, 15 \| 16, 16 \| 17, 17 \| 18, 18 \| 19, 19 \| 20, 20 \| 21, 21 \| 22, 22 \| 23, 23 \| 24, 24 \| 25, 25 |
|  | Please complete the LABORATORY AND OTHER TEST RESULTS form under the ADDITIONAL OUTCOMES event (Treatment Day 4, 8 and 14) |  |
|  | Please complete the LABORATORY AND OTHER TEST RESULTS form under the ADDITIONAL OUTCOMES event and the OUTCOME ASSESSMENT on the ADDITIONAL OUTCOMES event (Treatment Day 14) |  |
|  | You do not need to complete the LABORATORY AND OTHER TEST RESULTS form today. This form is completed on TREATMENT DAYS 4, 8 and 14. |  |
|  | Morning dose of tecovirimat taken? | 1, Yes \| 0, No |
|  | Morning dose of tecovirimat - dose (mg) | 1, 200mg \| 2, 400mg \| 3, 600mg \| 4, Other dose |
|  | Specify other dose amount in mg |  |
|  | This is a modified dose | 1, Yes |
|  | Reason for modification |  |
|  | Reason dose not taken | 1, Forgotten \| 2, Refused \| 3, Treatment complete \| 4, Other |
|  | State the reason not taken |  |
|  | Evening dose of tecovirimat taken? | 1, Yes \| 0, No |
|  | Evening dose of tecovirimat - dose (mg) | 1, 200mg \| 2, 400mg \| 3, 600mg \| 4, Other dose |
|  | Specify other dose amount in mg |  |
|  | This is a modified dose | 1, Yes |
|  | Reason for modification |  |
|  | Reason dose not taken | 1, Forgotten \| 2, Refused \| 3, Treatment complete \| 4, Other |
|  | State the reason not taken |  |
|  | Vaccinia Ig | 1, Yes \| 0, No |
|  | Date of vaccinia Ig |  |
|  | New smallpox vaccine | 1, Yes \| 0, No |
|  | Please keep the "Smallpox vaccination" section in the Baseline Assessment Form up to date |  |
|  | Other monkeypox treatment | 4, No / None \| 1, Brincidofovir \| 2, Cidofovir \| 3, Other |
|  | Please keep the "MPOX treatment" section in the Baseline Assessment Form up to date |  |
|  | Other monkeypox treatment name |  |
| **TESTS RESULTS** |  |  |
|  | Date of sample(s) |  |
| **Blood** | Blood - MPX PCR result | 1, Positive \| 0, Negative \| 2, Equivocal |
|  | Blood - Ct value |  |
| **Lesion Swab** | Lesion swab 1 - MPX PCR result | 1, Positive \| 0, Negative \| 2, Equivocal |
|  | Lesion swab 1 - Ct value |  |
|  | Lesion swab 2 - MPX PCR result | 1, Positive \| 0, Negative \| 2, Equivocal |
|  | Lesion swab 2 - Ct value |  |
| **Throat Swab** | Throat swab - MPX PCR result | 1, Positive \| 0, Negative \| 2, Equivocal |
|  | Throat swab - Ct value |  |
| **Other Swab** | Other Site swab - location | 1, Stool sample \| 2, Rectal swab \| 3, Vaginal swab \| 4, Urine \| 5, BAL \| 6, Ocular \| 7, Joint aspirate \| 8, Tissue sample \| 9, Lesion \| 10, Other |
|  | Please specify other location |  |
|  | Other Site swab - MPX PCR result | 1, Positive \| 0, Negative \| 2, Equivocal |
|  | Other Site swab - Ct value |  |
| **Pregnancy test** | Pregnancy test result | 1, Positive \| 0, Negative |
| **HIV test** | HIV test result | 1, Positive \| 0, Negative |
| **OUTCOME (At D14, D28, D60 and D180)** |  |  |
|  | Date of evaluation |  |
|  | Patient outcome | 1, All lesions are completely resolved and no serious complications \| 7, All lesions are no longer active and no serious complications \| 8, No lesions ever developed and no serious complications \| 2, One or more lesion(s) are active and no serious complications \| 3, Serious complication and/or hospitalisation due to monkeypox \| 4, Death \| 5, Early withdrawal \| 6, Loss to follow-up |
|  | Please ensure that the daily lesion assessment done on the same day is consistent with this outcome |  |
|  | PLEASE COMPLETE THE WITHDRAWAL FORM |  |
|  | Please complete a SAE form |  |
|  | First or earliest date on which the selected outcome was true |  |
|  | Reason for hospitalisation |  |
|  | Date of hospitalisation |  |
|  | Date of death |  |
|  | Cause of death |  |
|  | Reason for loss to follow-up: |  |
| **AE & SAE REPORT** |  |  |
|  | Date of notification |  |
|  | Event (please use MedDRA term) |  |
|  | Seriousness criteria of the event | 1, Not serious \| 2, Resulted in death \| 3, Life-threatening \| 4, Resulted in new or prolonged hospitalisation \| 5, Resulted in disability or incapacity \| 6, Resulted in congenital anomaly \| 7, Other event considered otherwise medically significant |
|  | Date of death |  |
|  | Date hospitalisation started or date from which hospitalisation was prolonged |  |
|  | Has the patient's hospitalisation ended? |  |
|  | Date hospitalisation ended |  |
|  | CTCAE grade of event | 1, 1 - Mild \| 2, 2 - Moderate \| 3, 3 - Severe \| 4, 4 - Life threatening \| 5, 5 - Death |
|  | Description of event |  |
|  | Start date of event |  |
|  | Date event became serious |  |
|  | Date site study team became aware of the event |  |
|  | Is the event still ongoing? | 1, Yes \| 0, No |
|  | Current status | 1, Recovering/improvement \| 2, Worsening |
|  | End date of event |  |
|  | Outcome of the event | 1, Resolved without sequelae \| 2, Resolved with sequelae \| 3, Continuing or not yet resolved \| 4, Death |
|  | Sequelae details |  |
|  | Actions taken for treatment of monkeypox | 1, No action taken \| 2, Dose reduced \| 3, Temporarily discontinued \| 4, Permanently discontinued \| 5, Not applicable - the participant wasn't receiving any treatment (i.e., anti-virals) for monkeypox |
|  | Other relevant documentation related to reported SAE  Please take care to anonymise uploaded files. |  |
| **Any Concomitant Medication at Time of Adverse Event** | Was the participant receiving any concomitant medication at the time of the event? | 1, Yes \| 0, No |
|  | Concomitant medication - Drug name |  |
|  | Concomitant medication - Dose |  |
|  | Concomitant medication - Units |  |
|  | Concomitant medication - Frequency |  |
|  | Concomitant medication - Start date |  |
| **Treatment Received for Adverse Event** | Did the participant receive any treatment for the event? | 1, Yes \| 0, No |
|  | AE treatment medication - Drug name |  |
|  | AE treatment medication - Dose |  |
|  | AE treatment medication - Units |  |
|  | AE treatment medication - Frequency |  |
|  | AE treatment medication - Start date |  |
|  | AE treatment medication - End date |  |
| **Causality - Site** | Relationship of the event to the study procedures (Site) | 1, Not related \| 2, Unlikely to be related \| 3, Possibly related \| 4, Probably related \| 5, Definitely related |
|  | Relationship of the event to monkeypox treatment (Site) | 1, Not related \| 2, Unlikely to be related \| 3, Possibly related \| 4, Probably related \| 5, Definitely related \| 6, Not applicable - the participant wasn't receiving any treatment (i.e., anti-virals) for monkeypox |
|  | Other causal relationship of event | 0, No other causal relationship \| 1, Progression of monkeypox disease \| 2, Medical condition \| 3, Concomitant medication \| 4, Other |
|  | Medical condition - please specify |  |
|  | Concomitant medication - please specify |  |
|  | Other - please specify |  |
| **Causality - Sponsor** | Relationship of the event to the study procedures (Sponsor) | 1, Not related \| 2, Unlikely to be related \| 3, Possibly related \| 4, Probably related \| 5, Definitely related |
|  | Relationship of the event to monkeypox treatment (Sponsor) | 1, Not related \| 2, Unlikely to be related \| 3, Possibly related \| 4, Probably related \| 5, Definitely related \| 6, Not applicable - the participant wasn't receiving any treatment (i.e., anti-virals) for monkeypox |
|  | Expectedness (Sponsor) | 1, Expected \| 2, Not expected \| 3, Not applicable |
| **Follow-up** | Add follow-up information? |  |
|  | Date of follow up (1) |  |
|  | Is the event still ongoing? |  |
|  | Seriousness criteria of the event | 1, Not serious \| 2, Resulted in death \| 3, Life-threatening \| 4, Resulted in new or prolonged hospitalisation \| 5, Resulted in disability or incapacity \| 6, Resulted in congenital anomaly \| 7, Other event considered otherwise medically significant |
|  | CTCAE grade of event | 1, 1 - Mild \| 2, 2 - Moderate \| 3, 3 - Severe \| 4, 4 - Life threatening \| 5, 5 - Death |
|  | Outcome of the event | 1, Resolved without sequelae \| 2, Resolved with sequelae \| 3, Continuing or not yet resolved \| 4, Death |
|  | End date of event |  |
|  | Relevant information related to the follow-up of the SAE |  |
|  | Other relevant documentation related to the follow-up of the SAE  Please take care to anonymise uploaded files |  |
|  | Add another follow-up update? |  |
|  | Date of follow up (2) |  |
|  | Is the event still ongoing? |  |
|  | Seriousness criteria of the event | 1, Not serious \| 2, Resulted in death \| 3, Life-threatening \| 4, Resulted in new or prolonged hospitalisation \| 5, Resulted in disability or incapacity \| 6, Resulted in congenital anomaly \| 7, Other event considered otherwise medically significant |
|  | CTCAE grade of event | 1, 1 - Mild \| 2, 2 - Moderate \| 3, 3 - Severe \| 4, 4 - Life threatening \| 5, 5 - Death |
|  | Outcome of the event | 1, Resolved without sequelae \| 2, Resolved with sequelae \| 3, Continuing or not yet resolved \| 4, Death |
|  | End date of event |  |
|  | Relevant information related to the follow-up of the SAE |  |
|  | Other relevant documentation related to the follow-up of the SAE  Please take care to anonymise uploaded files |  |
|  | Add another follow-up update? |  |
|  | Date of follow up (3) |  |
|  | Is the event still ongoing? |  |
|  | Seriousness criteria of the event | 1, Not serious \| 2, Resulted in death \| 3, Life-threatening \| 4, Resulted in new or prolonged hospitalisation \| 5, Resulted in disability or incapacity \| 6, Resulted in congenital anomaly \| 7, Other event considered otherwise medically significant |
|  | CTCAE grade of event | 1, 1 - Mild \| 2, 2 - Moderate \| 3, 3 - Severe \| 4, 4 - Life threatening \| 5, 5 - Death |
|  | Outcome of the event | 1, Resolved without sequelae \| 2, Resolved with sequelae \| 3, Continuing or not yet resolved \| 4, Death |
|  | End date of event |  |
|  | Relevant information related to the follow-up of the SAE |  |
|  | Other relevant documentation related to the follow-up of the SAE  Please take care to anonymise uploaded files |  |
|  | Add another follow-up update? |  |
|  | Date of follow up (4) |  |
|  | Is the event still ongoing? |  |
|  | Seriousness criteria of the event | 1, Not serious \| 2, Resulted in death \| 3, Life-threatening \| 4, Resulted in new or prolonged hospitalisation \| 5, Resulted in disability or incapacity \| 6, Resulted in congenital anomaly \| 7, Other event considered otherwise medically significant |
|  | CTCAE grade of event | 1, 1 - Mild \| 2, 2 - Moderate \| 3, 3 - Severe \| 4, 4 - Life threatening \| 5, 5 - Death |
|  | Outcome of the event | 1, Resolved without sequelae \| 2, Resolved with sequelae \| 3, Continuing or not yet resolved \| 4, Death |
|  | End date of event |  |
|  | Relevant information related to the follow-up of the SAE |  |
|  | Other relevant documentation related to the follow-up of the SAE  Please take care to anonymise uploaded files |  |
| **PREGNANCY FOLLOW UP** |  |  |
|  | Has the mother consented to pregnancy follow-up? |  |
|  | Date of pregnancy test |  |
|  | Date of last menstruation |  |
|  | Expected delivery date |  |
|  | Pregnancy outcome | 1, Baby is in good health \| 2, Congenital malformations requiring admission to the neonatal unit \| 3, Abortion (by choice) \| 4, Abortion (for medical reasons) \| 5, Miscarriage \| 6, Stillbirth \| 7, Neonatal death \| 8, Maternal death |
|  | Date of outcome |  |

## **Appendix 2 – Summary of responsible ethics committees and respective reference numbers**

| Country | Ethics Committee | Reference number |
| --- | --- | --- |
| Belgium | Federal Agency for Medicines and Health Products | 2022-501132-42-00 |
| France | Comité de protection des personnes Ile De France III | 2022-501132-42-00 |
| Italy | Agenzia Italiana del Farmaco | 2022-501132-42-00 |
| Netherland | Central Committee on Research Involving Human Subjects | 2022-501132-42-00 |
| Spain | Agencia Española de Medicamentos y Productos Sanitarios | 2022-501132-42-00 |
| Switzerland | Commission cantonale d'éthique de la recherche | 2022-01075 |
| UK | East of England - Cambridge East Research Ethics Committee | 22/EE/0143 |

## **Appendix 3 – MPXV recommendations and practices in MOSAIC participants Countries**

| **BELGIUM** |
| --- |
| **National recommendations** |
| **Indications:**  Antivirals may be considered within clinical studies or compassionate use protocols, particularly for patients with severe symptoms or who may be at risk of poor clinical outcome, such as immunocompromised subjects. Post-exposure vaccination (ideally within four days of exposure) may be considered for high risk contacts of a confirmed case such as healthcare professionals, including laboratory personnel, after careful evaluation of the risks and benefits.  Only available for patients hospitalized with severe Monkeypox. Limited treatment available. Every treatment allocation must be discussed with 2 experts from our Task Force.  **Medicines:**  -Antivirals: Tecovirimat  -Vaccines: Imvanex  Reference : <https://www.health.belgium.be/sites/default/files/uploads/fields/fpshealth_theme_file/20220614_shc_9720_monkeypox_vaccination_vweb.pdf> |
| **Tecovirimat availability** |
| 10 doses available for the country, limited to immunodepressed patients or patients with a severe cinical presentation. Tecovirimat was available in a very restricted quantity in Belgium as of September 2, 2022. Only patients hospitalized with mpox were eligible for the treatment after the green light was given from a consensus amongst 2 infectious diseases specialists. |
| **Did the clinicians follow the guidelines?** |
| Clinicians followed the guidelines |

| **FRANCE** |
| --- |
| **National recommendations** |
| **Indications:** Yes, for severe presentations or patients with risk factors for severity (immunocompromised patients, pregnant women and children)  Main indications in practice  - Ophthalmological form (conjunctivitis, keratitis)  - Severe cutaneous form requiring surgery  - Form suggestive of vasculitis (purpura, lymphangitis)  - Diffuse cutaneous form in an immunocompromised patient (transplant, immunosuppressive treatment, HIV < 200 CD4)  - Pregnant women  - Myocarditis, encephalitis  **Medicines:** Tecovirimat in first line then Brincofovir then Cidofovir or Vaccine Ig  Reference : <https://www.hcsp.fr/Explore.cgi/avisrapportsdomaine?clefr=1212> |
| **Tecovirimat availability** |
| ***First supply in July 2022 :*** *Available and provided doses after authority agreement, for patients with a severe clinical presentation*  ***August 2022*** *: (In some hospitals) Provision of an emergency stock of Tecovirimat for patients meeting the main indication mentioned above, for other presentation (e.g : Complication of proctitis) green light from authority needed before treatment initiation.* |
| **Did the clinicians follow the guidelines?** |
| Yes, they did |

| **ITALY** |
| --- |
| **National recommendations** |
| **Indications:** Antivirals may be considered within clinical studies or compassionate use protocols, particularly for patients with severe symptoms or who may be at risk of poor clinical outcome, such as immunocompromised subjects. Post-exposure vaccination (ideally within four days of exposure) may be considered for high risk contacts of a confirmed case such as healthcare professionals, including laboratory personnel, after careful evaluation of the risks and benefits.  **Medicines:** Antivirals and type of Vaccines are not specified.  There is only one document from the Italian Minister of Health published in August 2022 where a few lines reported that tecovirimat could be used in immunocompromised patients or in RCT.  No further communication after that. This means that in Italy, the only way to use tecovirimat was as compassionate through AIFA. Probably a very few immunocompromised patients were treated. |
| **Tecovirimat availability** |
| 25 March 2022 – Drug approved by the Italian Medicines Agency (AIFA). A few hundreds treatments available. No supply limitations. A few patients treated according to the recommendations cited above from the Minister of Health. |
| **Did the clinicians follow the guidelines?** |
| Yes, they did. |

| **SPAIN** |
| --- |
| **National recommendations** |
| In Spain they was a very limited supply of Tecovirimat.  It can be requested through a "special use protocol" only for hospitalized patients with neumonitis, neurological complications or lymphadenopathies compromising airway. Also for immunosuppressed. No recommendations for Brincidofovir. Cidofovir is being used by dermatologist to treat some skin lesions topically.  They were 3 levels of priority   - ​Priority 1: pneumonia. Encephalitis or meningoencephalitis. Corneal ulcers or other eye injuries with a risk of permanent sequelae that affect vision. Pharyngeal lesions that prevent the swallowing of liquids and/or present total or partial compromise of the airway. - Priority 2: severe proctitis. Severe cellulitis or with a risk of permanent sequelae (functional compromise of the penis). Immunocompromised patients with persistent fever or disseminated disease with generalized involvement. - Priority 3: all other patients infected with monkeypox who do not have a contraindication.   Reference: <https://www.sanidad.gob.es/profesionales/saludPublica/ccayes/alertasActual/alertaMonkeypox/docs/20220610_ProtocoloMPX.pdf> |
| **Tecovirimat availability** |
| 25MAY22, 10-14 doses available for the country, initially, it was limited to patients with neurological involvement, lung involvement, ocular/corneal involvement, respiratory tract lesions that prevent swallowing and/or breathing, and signs of shock.  The arrival of more drugs was gradual and this managed to modify, expand and make the administration criteria more flexible. |
| **Did the clinicians follow the guidelines?** |
| The clinicians have followed the guidelines because a committee of experts was organised to evaluate the requests for treatment made by the doctors who diagnosed the cases of mpox. With the consensus of this committee and always with scientific evaluation and criteria, the advisability of administering the treatment was decided, a decision that could be modified with the clinical evolution of the patient. |

| **SWITZERLAND** |
| --- |
| **National recommendations**  With regards to the current limitations of the drugs in terms of availability in Switzerland and/or route of administration, a treatment course can be considered in the following situations (low level of evidence):  1. Patients at high risk for severe disease, such as  a. immunocompromised individuals (transplant patients, patients with HIV infection and CD4 cell count below 200 CD4/mm3, on immunosuppressive drugs),  b. pregnant women and children (especially below 8 years-old). A gynecologist/obstetrician as well as pediatric infectious disease specialist should always be consulted in such situations.  2. Patients with a severe disease presentation for example:  a. Patients presenting with more than 100 lesions (severe) or more than 250 lesions (very severe).  b. Patients with lesions leading to functional inability (throat or genital mucosal lesion, eyes) or uncontrollable discomfort (uncontrolled pain on opoids).  3. Patients hospitalized with organ dysfunction (encephalitis, myocarditis, sepsis) or hemorrhagic lesions, or evocative form of vasculitis (purpura).  The first line of treatment is oral tecovirimat due to its better in vitro performance and better risk profile when compared to other direct antivirals. Tecovirimat is currently available in Switzerland for compassionate use and/or within the MOSAIC study. Access to tecovirimat in Switzerland is currently restricted within the framework of a collegial and multidisciplinary decision in a validation committee.  IV cidofovir is licensed and available in Switzerland (off label for monkeypox).  We suggest prioritizing oral tecovirimat when considering antiviral treatment in some clinical situations described below.  **Reference :**  <https://www.google.com/url?sa=t&rct=j&q=&esrc=s&source=web&cd=&cad=rja&uact=8&ved=2ahUKEwjrmvq3kaH-AhVdgf0HHckAAacQFnoECA0QAQ&url=https%3A%2F%2Fwww.bag.admin.ch%2Fdam%2Fbag%2Fde%2Fdokumente%2Fmt%2Fp-und-p%2Feksg%2Fmonkeypox-treatment-recommandations-ssi-fcsti.pdf.download.pdf%2Fmonkeypox-treatment-recommandations-ssi-fcsti.pdf&usg=AOvVaw0ADHajY2NP0J9YDdQB0bGD> |
| **Tecovirimat availability** |
| Tecovirimat was available from the 11^th^ of August 2022 in Switzerland. The country was supplied only with 96 bottles (48 treatments) from SIGA, hence the necessity for clear recommendations regarding Tecovirimat prescription. |
| **Did the clinicians follow the guidelines?** |
| Yes – all but one of those on tecovirimat have been included in MOSAIC. |

| **UNITED KINGDOM** |
| --- |
| **National recommendations** |
| In the UK, tecovirimat was prescribed to inpatients according to nationally agreed criteria, to facilitate gate-keeping of a limited national supply held at my hospital. This is what was agreed early on and followed throughout (treating around 60 hospitalised cases with tecovirimat):  Symptomatic with a syndrome compatible with *ongoing* monkeypox virus infection and meeting any one or more of the criteria for severe or complicated disease as outlined below:   - critical illness where monkeypox virus infection is considered to be a key factor driving the critical condition of the patient - intractable pain - rectal abscess or fistula formation - upper respiratory tract mucocutaneous involvement that is affecting swallowing or airways - patient with primary or acquired immunodeficiency, or on immunosuppressive medication as per Green Book [UK vaccination guidelines] definitions - ocular or periocular disease - encephalitis, meningitis or other neurological manifestation - extensive cutaneous disease (for example more than 100 lesions) - complex genital disease: difficulty passing urine due to swelling or lesions causing direct urinary obstruction   “Treatment outside the above “severe” criteria may be used in the context of treating children or to facilitate shortening the duration of infectiousness due to other complex medical needs. Such treatment must be considered and agreed by the appropriate multidisciplinary team.” One case was was treated according to this criteria: a patient was given treatment to hopefully shorten the period of active infection and the in-hospital hemofiltration required when safe outpatient dialysis for their chronic kidney disease was impossible.   Only inpatients were treated with tecovirimat in the UK (separate to the Platinum RCT for outpatients) and while sicker overall than community patients, most of our hospital cases (around 180 in total) did not receive tecovirimat – we just managed their pain and treated their secondary bacterial infections etc.  Reference: https://www.england.nhs.uk/commissioning/publication/tecovirimat-as-treatment-for-patients-hospitalised-due-to-monkeypox-virus-infection/ |
| **Tecovirimat availability** |
| Tecovirimat was available in the UK throughout the 2022 outbreak for treating hospitalised cases who met the agreed criteria for treatment. |
| **Did the clinicians follow the guidelines?** |
| YES |

1. Monolix 2024R1 LS, a Simulations Plus company. [↑](#footnote-ref-2)
2. Suñer C, Ubals M, Tarín-Vicente EJ, Mendoza A, Alemany A, Hernández-Rodríguez Á, et al. Viral dynamics in patients with monkeypox infection: a prospective cohort study in Spain. The Lancet Infectious Diseases. 2023;23(4):445-53. [↑](#footnote-ref-3)
